# Recurrent symmetrical bendings cause dwarfing in *Hydrangea* through spatial molecular regulation of xylem cell walls

**DOI:** 10.3389/fpls.2023.1268272

**Published:** 2024-01-16

**Authors:** Béra Ley-Ngardigal, Hanaé Roman, Nathalie Brouard, Lydie Huché-Thélier, Vincent Guérin, Nathalie Leduc

**Affiliations:** ^1^ Univ Angers, Institut Agro, INRAE, IRHS, SFR QUASAV, Angers, France; ^2^ Hortensia France Company, Rives-du-Loir-en-Anjou, France

**Keywords:** biomimetics, mechanical stress, non-chemical dwarfing, ornamental, plant compactness, stem flexure, thigmomorphogenesis, wood anatomy

## Abstract

Environmental prejudices progressively lead to the ban of dwarfing molecules in agriculture, and alternatives are urgently required. Mechanical stimulation (MS) is a promising, eco-friendly, and economical technique, but some responses to mechanical stimulation vary from one plant species to another. Additionally, as more frequent and violent wind episodes are forecasted under global climate change, knowledge of plant responses to stimuli mimicking wind sways is decisive for agriculture. However, little is known about plant mechanosensitive responses after long-term, recurrent MS. Here, the effects of 3-week, recurrent, symmetrical bendings (1 or 12 per day) in *Hydrangea macrophylla* stems are examined. Bendings repressed internode elongation and leaf area development, whereas the diametrical growth of the basal internode is increased. Responses were dose-dependent, and no desensitization was observed during the 3 weeks of treatment. MS was almost as efficient as daminozide for plant dwarfing, and it improved stem robustness. Histological and molecular responses to MS were spatially monitored and were concordant with ongoing primary or secondary growth in the internodes. Our molecular data provide the first knowledge on the molecular paths controlled by mechanical loads in *Hydrangea* and revealed for the first time the involvement of *XYP1* in thigmomorphogenetic responses. MS still had a transcriptional impact 48 h after the last bending session, promoting the expression of *XYP1*, *FLA11*, and *CAD1* while repressing the expression of *EXP3* and *XTH33* homologs in accordance with xylogenesis, cell wall thickening, and lignin deposition in the xylem of basal internodes. In upper elongating internodes, repression of *XYP1*, *CAD1*, *SAMS1*, and *CDC23* homologs is correlated with ongoing primary, even though stunted, growth. For producers, our findings highlight the potential of MS as a sustainable and economical option for controlling plant compactness in *Hydrangea* and show valuable reinforcement of stem strength.

## 1 Introduction

In ornamental crops such as *Hydrangea*, reduction of shoot length and increased branching contribute to the production of high-quality, compact, and heavily flowered plants that meet markets’ and consumers’ criteria (Boumaza et al., 2010; Megersa et al., 2018). These criteria are classically obtained after repetitive chemical treatments using plant growth regulators (PGRs), especially growth retardants (Dilta et al., 2015; Rademacher, 2015; Jamwal et al., 2018). Gibberellin biosynthesis inhibitors, such as daminozide, are the most widely used growth retardants in plant production, as they account for approximately 40% of the global PGR market (Rademacher, 2017). However, several studies revealed the negative impacts of these chemicals on both humans and the environment, which has led public authorities to progressively phase out growth retardant use in horticultural industries (Andersen and Andersen, 2000; Sorensen and Danielsen, 2006; Bergstrand, 2017). This regulation strongly impacts the ways ornamental plants are produced today (Carvalho et al., 2008). Luckily, numerous studies have shown that environmental factors have an impact on plant compactness (Huché-Thélier et al., 2011; Morel et al., 2012; Alem et al., 2015; Li-Marchetti et al., 2015; Demotes-Mainard et al., 2016; Huché-Thélier et al., 2016; Bergstrand, 2017; Crespel et al., 2018; Runkle et al., 2019), and these responses could be exploited to develop non-chemical-based dwarfing methods. This strategy is underway and has brought some success in several species, but it remains an urgent challenge to overcome for most crop productions.

Mechanical stimulation (MS) is one of these alternative methods (Börnke and Rocksch, 2018), and it has been inspired by the thigmomorphogenetic effects of winds on plants (Jaffe, 1973). Wind sways impose recurrent mechanical loads on plants, which can be uni- or multi-directional, of variable intensities and durations, depending on several factors such as location and topography (de Langre, 2008; Gardiner et al., 2016). These characteristics are different from the long-lasting unidirectional mechanical loads imposed by gravity on leaned stems, with these phenomena already being well documented (Moulia and Fournier, 2009; Ramos et al., 2012; Gardiner et al., 2014). Thigmomorphogenesis under wind sways is often characterized by a reduction of shoot elongation together with an enhancement of diametrical growth (Neel and Harris, 1971; Jaffe and Forbes, 1993). This leads to the development of smaller plants with larger stems more capable of withstanding strong winds and maintaining their vertical position (Bonnesoeur et al., 2016; Gardiner et al., 2016). Often, this is accompanied by an increased branching and a reduction of the global leaf area (Vernieri et al., 2003; Morel et al., 2012). All these characteristics meet in fact the goals of the ornamental industry (Andersen and Andersen, 2000). In addition, MS sometimes induces a delay in flowering time, as observed in *Arabidopsis thaliana* (Lange and Lange, 2015), *Helianthus annuus* (Goodman and Ennos, 1996), and *Brachypodium distachyon* (Gladala-Kostarz et al., 2020).

Several techniques were developed to mimic wind effects on plants for either research purposes or plant production: shaking plants under air streams or using a vibrating table, bending stems by hand, or through recurrent passing of a material, such as a solid bar or with a softer material. So far, these techniques were applied to a few ornamental species in production, such as *Salvia splendens* (Vernieri et al., 2003; Mensuali-Sodi et al., 2006), *Callistephus chinensis* (Autio et al., 1994), *Petunia x atkinsiana* (Jędrzejuk et al., 2020), and *Impatiens capensis* (Anten et al., 2009). In rosebush, for example, MS performed using a solid bar effectively reduced stem elongation by up to 23% in some varieties (Crespel et al., 2018). Nevertheless, thigmomorphogenetic responses strongly depend on plant genotype and the plant’s herbaceous, climbing, or woody growth habit (Börnke and Rocksch, 2018). They also depend on the mechanical load intensities, frequencies, and directions (de Langre, 2008; Niez et al., 2020).

Mechanisms behind wind-induced thigmomorphogenesis have been less investigated compared to gravimorphic mechanisms (Niez et al., 2020). To this day, investigations have been only dealing with very few species, mainly *Pinus taeda* (Telewski and Jaffe, 1986; Dranski et al., 2018), *Abies fraseri* (Telewski, 1989), poplars and their hybrids (Kern et al., 2005; Fluch et al., 2008; Niez et al., 2019; Niez et al., 2020), *A. thaliana* (Braam and Davis, 1990; Antosiewicz et al., 1997; Johnson et al., 1998; Paul-Victor and Rowe, 2011; Zhdanov et al., 2021), *Solanum lycopersicum* (Gartner, 1994; Cipollini and Redman, 1999; Coutand et al., 2000), and more recently *B. distachyon* (Gladala-Kostarz et al., 2020; Coomey et al., 2021). Thus, research needs to broaden its range to a larger plant panel in order to decipher the entire machinery behind the responses to this mechanical stimulus.

Histological investigations revealed that within the woody stems of *Pinus*, *Abies*, and *Populus* plants that have been subjected to non-static swaying, due to transient symmetrical or asymmetrical unidirectional bendings, a particular wood, called flexure wood (Telewski, 1989), develops itself in the bent stems, improving its tolerance to mechanical loads and maintaining verticality while diminishing mechanical failures (Telewski, 2016; Roignant et al., 2018; Niklas and Telewski, 2022). When unidirectional mechanical load is applied, flexure wood sometimes develops asymmetrically, displaying an elliptical shape on stem cross-sections in the direction of bending (Telewski, 1989; Telewski, 2016; Roignant et al., 2018). However, this does not occur in all species; therefore, these opposite responses need to be further investigated (Telewski, 1989). In poplar, which is the most investigated woody angiosperm on this subject, significant reductions of vessel lumen areas, vessel diameters, and vessel frequencies in response to repeated bi-directional flexures (backward and forward) were observed in the flexure wood, and the development of wood fibers with no cellulosic G-layer was also reported (Kern et al., 2005). Interestingly, in this same species, unilateral bending caused radically different responses. In the stretched side of the stem, differentiation of up to one-third of wood fibers with a cellulosic G-layer occurred, while in the compressed side of the stem, there is almost no observed development of wood fibers containing such G-layer (Roignant et al., 2018). These results illustrate how plants may respond differently and adapt according to the characteristics of the mechanical load they encounter, in particular the contrast between symmetrical and asymmetrical bendings.

At the molecular level, there is very little information about the mechanisms involved in the perception and responses of the stem to recurring mechanical loads mimicking wind impacts. Most molecular data were about hybrid poplar after one or two asymmetrical bendings (Martin et al., 2010; Pomiès et al., 2017). In correlation with the histological impact of MS on stems, several cell wall genes were identified as responsive within a few hours after stimulation. For example, upregulation of the closest homologs of touch gene *TCH4* occurs after one single or two asymmetrical bendings of the poplar stem (Martin et al., 2010). *TCH4* is a member of the XET/XTH family encoding xyloglucan endotransglycosylase/hydrolases involved in the control of cell wall plasticity (loosening or thickening) in secondary xylem cells of *Arabidopsis* (Xu et al., 1995; Kushwah et al., 2020). This upregulation correlates well with an accumulation of *TCH4*-encoded XET protein in the epicotyl pith parenchyma cells, hypocotyl epidermis, and primary xylem in *Arabidopsis* subjected to 4 days of continuous blowing (Antosiewicz et al., 1997). Other genes encoding parietal proteins, such as *CELLULOSE SYNTHASE* and *FASCICLIN-LIKE ARABINOGALACTAN PROTEINS*, and some *PECTINASES*, including *POLYGALACTURONASES* and *PECTIN ESTERASES*, were also upregulated after a single asymmetrical flexure in poplar stem, while some such as the cell wall expansion gene *EXPANSIN B3* were downregulated (Pomiès et al., 2017). In the same study (Pomiès et al., 2017), the upregulation of genes involved in lignin biosynthesis such as *CINNAMOYL ALCOHOL DEHYDROGENASE* and *S-ADENOSYL-L-METHIONINE SYNTHETASE* were also reported. Indeed, the latter is involved in the synthesis of *S*-adenosyl-l-methionine, an important methyl donor during the biosynthesis of both G- and S-type lignin units (Li et al., 2022). In addition, repression of the expression of the *CELL DIVISION CYCLE* gene within the first 30 minutes following stimulation was measured and correlated with the transient cessation of cambial growth observed during the first hours after one bending prior to the resumption of secondary growth (Pomiès et al., 2017).

When plants are subjected to recurrent mechanical stimulation, attenuation of the thigmomorphogenic responses is often observed, and this phenomenon may prevent unnecessary reduction of growth while still allowing plants to resist (Martin et al., 2010; Pomiès et al., 2017). For example, in poplar, 96% of the early mechanoresponsive genes showed reduced response to a second bending applied 24 h after the first one (Pomiès et al., 2017). Despite the importance of understanding plant’s long-term responses to MS under windy natural environments and in horticultural crops, knowledge of the mechanisms behind such desensitization reactions is still in its infancy (Brenya et al., 2022). Only two recent studies report molecular control after long-term recurrent mechanical stimulations of stems. First, in hybrid poplar, a link was established between the cell wall modifications in flexure wood after 8 weeks of recurrent asymmetrical bending of the stem and the expression of cell wall formation genes in stems (in particular *FASCICLIN-LIKE ARABINOGALACTAN PROTEINS*) (Roignant et al., 2018). Second, in *B. distachyon*, a monocotyledon where no cambial secondary growth occurs, a recent report links the inactivation of gibberellin responses with *SECONDARY WALL INTERACTING bZIP* transcription factors and secondary wall thickening in roots after 3 weeks of daily mechanical bending of the plant (Coomey et al., 2021).

With the aim to broaden knowledge and evaluate the genericity of reported plant responses to recurrent long-term mechanical loads and desensitization, we investigated the responses of *Hydrangea macrophylla*, an important ornamental bush species, making it the only woody angiosperm species after poplar to be examined from plant to molecular levels after wind-mimicking MS. We focused on histological changes through analyses of stem cross-sections and quantitative expressions of nine MS-responding genes that were already described in literature (Martin et al., 2010; Pomiès et al., 2017) as related to cell wall and cell growth. We also observed for the first time that *XYLOGEN PROTEIN 1* (*XYP1*), a gene encoding an arabinogalactan protein that promotes xylem cell differentiation (Motose et al., 2004; Ma et al., 2014), is responsive to MS. We carried out our analysis on two portions of a stem, allowing the simultaneous comparison of MS responses in primary and secondary growing internodes along the same stem. We carried out measurements 3 weeks of recurrent daily MS treatment using two frequencies of MS in order to evaluate quantitative growth and desensitization responses according to the number of daily MS. Finally, in a perspective of replacement and/or reduction of growth retardants use in horticulture, we compared the efficiencies of MS and daminozide, as it is the most widely used PGR for plant compactness, and we discussed the potential benefit of MS in *H. macrophylla* production.

## 2 Materials and methods

### 2.1 Plant material

*H. macrophylla* cv. ‘Wudu^®^’ plants from a single genotype were produced from micro-cuttings (Galopin et al., 1996) by Hortensia France company (Rives-du-Loir-en-Anjou, France). When the root system of the micro-cuttings had completely filled the micro-plugs (Green Products, Jongkind Substrates, Aalsmeer, Netherlands; 35% Swedish peat, 27.5% brown peat, and 37.5% perlite) they were inserted into, they were transferred into 10-cm-diameter pots containing a mixed fertilized peat–coconut substrate (composition for 1 m^3^: 750 L peat, 250 L coconut fiber, 0.70 kg Pg-Mix Haifa 12-14-25, 0.3 kg microelements, 0.70 kg limestone, 1 kg dolomite lime, and 5 kg clay). At the beginning of each experiment, young plants developed three pairs of well-developed leaves from a single unbranched stem (1.3-cm length and 2.6-cm diameter at the collar on average) composed of three internodes (see **Supplementary Material** for illustration). MS experiments were carried out in a growth chamber (T° day/night, 23/21°C; relative humidity (RH) 80%) where plants were sub-irrigated once a week with a nutrient solution (Angibaud-Soluveg^®^ ALC 47; electrical conductivity (EC), 1.8 mS/cm; pH 6). Plants were grown under a photoperiod of 16 h/day under artificial light using light-emitting diodes (Topband, Shenzhen, China) with a photon flux density [380–780 nm] of 100 ± 2 µmol·m^−2^·s^−1^. At the end of the experiments, some plants were further grown until flowering.

### 2.2 Mechanical stimulation

MS device, as described in Morel et al. (2012), was composed of two arms, allowing simultaneous mechanical treatments, with each arm moving backward and forward at a speed of 12 m/h over a 2.1-m^2^ surface area (2.1 m length × 1.0 m width). An automatic launch program allowed to tune days, frequencies, time slots, and duration of the stimulations. The material used for stimulation was an unfringed 2-mm-thick plastic curtain (2.3 kg/m^2^), which did not induce leaf damage or uprooted *H. macrophylla* young plants (Ley-Ngardigal et al., 2023). The height of the stimulating material was adjusted along the experiment so that the lower part of the curtain always reached the plants 0.5 cm below their apex. Upon each MS, the stem was bent at an angle of 30° with reference to verticality for approximately 30 s (see **Supplementary Material**). MS treatment was applied for 3 weeks, each morning between 9:00 a.m. and 10:00 a.m., for five consecutive days per week. In one bending treatment, plants were mechanically stimulated by one single bending per day (one forward the first day, one backward the second day, and so on until the last day). In 12 bending treatments, plants were mechanically stimulated by 12 successive bendings (i.e., six forward and six backward) each day. Each mechanical stimulation experiment was carried out three times (i.e., three biological replicates), and each biological replicate comprised 15 experimental plants that were measured and 20 border plants (i.e., plants that were not measured and placed around the 15 experimental plants); thus a total of 3 × 15 plants were measured per experiment. All plants were placed under each arm of the MS device.

### 2.3 Treatment with chemical growth retardant

In the same way as the mechanical stimulation experiments (see “Mechanical stimulation” section), 3 × 15 plants were measured after being treated once with daminozide, a chemical growth retardant (Dazide ENHANCE^®^, 5 g/L), at the same developmental stage as the plants at the beginning of MS experiments. These chemically dwarfed plants (named PGR treatment) were grown under the same environmental conditions as MS plants and control plants (i.e., non-MS and non-chemically dwarfed plants) for 3 weeks.

### 2.4 Stem ovalization

To compare the effects of recurrent bilateral MS (see “Mechanical stimulation” section) with permanent, static flexure on the ovalization of *H. macrophylla* stem, the stems of a total of six plants (two plants per biological repetition) were tied permanently at an angle of 45° with reference to verticality during three consecutive weeks with no interruption. Approximately 55-µm cross-sections of the most basal internode at the collar and of the sixth internode (the last developed internode at the beginning of the experiment) were hand-cut using a razor blade. Cross-sections were stained using Mirande’s reagent and observed using a Zeiss Axio Zoom V16 Macroscope (2019) (Carl Zeiss, Oberkochen, Germany; see “Histological analysis” section). At the end of the experiment, two distinct stem diameters were measured: one in the bending direction (parallel with the bends, *D*_//_) and the other perpendicular to the bending direction (*D*_⊥_). Ovalization of the stem cross-sections was determined as follows according to Niez et al. (2019):


$$\text{Ovalization }=\;100\times \;\frac{D_{//}\;-\;D_{⊥}}{D_{⊥}}.$$

The results obtained from this experiment are shown in the **Supplementary Material**.

### 2.5 Whole plant measurements

Plant stem height was measured between the stem collar and below the apex using a ruler with a 0.5-mm accuracy 1 day prior to MS treatment and once a week on days 7, 14, and 21. At the beginning and end of each experiment, stem diameter at the collar was measured using a caliper with a 0.01-mm accuracy as well as the number of pairs of leaves and internodes. At the end of each experiment, the leaves of five randomly chosen plants were scanned, and leaf areas were determined using ImageJ image processing software (version 1.52 p).

### 2.6 Histological analysis

Cross-sections of approximately 55-µm thickness of the most basal internode at the collar (internode 1) and in the middle of upper internode 3 were hand-cut using a razor blade. Cross-sections were stained using the following: 1) Mirande’s reagent to differentiate lignified tissues (green-blue staining) from cellulosic tissues (pink staining) (Mondolot, 2001) and 2) Wiesner’s reagent to quantify lignified tissues appearing red/pink (Nakano and Meshitsuka, 1992; Ferreira et al., 2017). For Mirande’s reagent, cross-sections were first bathed in sodium hypochlorite 9.6% for 15 minutes and then in distilled water for 3 minutes, followed by an acetic acid 20% bath for 5 minutes. Sections were then stained with Mirande’s reagent (a mix of green iodine and carmine alum; Sordalab, Etampes, France) for 15 minutes and rinsed in distilled water for 3 minutes. For Wiesner’s reaction, cross-sections were successively immersed in baths of sodium hypochlorite 9.6% for 10 minutes, distilled water for 3 minutes, phloroglucinol 2% for 10 minutes, hydrochloric acid 37% for a few seconds, and distilled water to stop immediately the reaction. Cross-sections were then observed using a Zeiss Axio Zoom V16 Macroscope (2019). The software ImageJ and the color clustering plugin were used to analyze the images. For all the images, the red, blue, and green channels and the SimpleKMeans method with the default settings of the color clustering plugin (Hassan et al., 2017) were used. Cell wall structures were also observed using scanning electron microscopy. Hand cross-sections (approximately 55 µm thick) were made using a razor blade in basal internode 1 and immediately immersed in distilled water to prevent drying. Cross-sections were carefully drained, then placed on a carbon disk, and quickly observed under a Phenom G2 PRO desktop scanning electron microscope (SEM). Image analyses were made using Phenom G2 PRO SEM software.

### 2.7 Phylogenetic analysis of candidate genes

Identification of *H. macrophylla* sequences was performed by similarity comparison of *A. thaliana*, *S. lycopersicum*, *Populus trichocarpa*, *Camellia sinensis*, and *Oryza sativa* protein sequences (all available on https://www.ncbi.nlm.nih.gov/). The accession number of sequences used in this paper is available in the **Supplementary Data** (see **Supplementary Material**). Multiple sequence alignments were performed using the default parameters of ClustalW in MEGAX^®^ software (Kumar et al., 2018). Phylogenetic trees were inferred using the neighbor-joining method (Saitou and Nei, 1987) and were assessed by bootstrapping (1,000 replicates) using MEGAX^®^ software. Ten genes involved in phenylpropanoid pathway were identified: *HmSAMS1* (*S-ADENOSYL-L-METHIONINE SYNTHETASE 1*) in lignin biosynthesis, *HmCAD1* (*CINNAMYL ALCOHOL DEHYDROGENASE 1*) in cell division, *HmCDC23* (*CELL DIVISION CYCLE 23*) in xylem differentiation, *HmXYP1* (*XYLOGEN PROTEIN 1*) in cell wall modification, *HmCESA5* (*CELLULOSE SYNTHASE 5*), *HmFLA11* (*FASCICLIN-LIKE ARABINOGALACTAN-PROTEIN 11*), *HmEXPB3* (*β-EXPANSINE 3*), *HmPE1* (*PECTINE ESTERASE 1*), *HmPGX3* (*POLYGALACTURONASE INVOLVED IN EXPANSION 3*), and *HmXTH33* (*XYLOGLUCAN : XYLOGLUCOSYL TRANSFERASE 33*). Phylogenetic trees are shown in the **Supplementary Material**.

### 2.8 RNA extraction, cDNA synthesis, and RT-qPCR

Following 2 weeks of 12B mechanical treatment, basal internode 1 and upper internode 3 from 45 plants (15 plants per biological repetition) were collected 48 h after the last mechanical stimulation, i.e., at 10 a.m. on the second day with no mechanical treatment of that week. Approximately 60 mg of each internode type was crushed in a mortar using liquid nitrogen. RNAs were extracted using an RNA plant extraction kit (Macherey-Nagel, Düren, Germany). The quality and quantity of RNAs were checked using Nanodrop One (Thermo Scientific, Waltham, MA, USA), and their integrity was verified on an agarose gel. From 500 ng RNA, the synthesis of complementary DNA (cDNA) was carried out using the iScript Reverse Transcription Supermix for RT-qPCR kit (Bio-Rad, Hercules, CA, USA). RT-qPCR was carried out in a final volume of 15 µL containing 3 µL of cDNAs diluted to 1/50, 1 µL of primer pairs, 4 µL of SYBR Green Supermix (Bio-Rad), and 7 µL of ultrapure water. The following amplification program was used: initiation at 98°C for 3 s, 40 cycles including a denaturation step at 95°C for 5 s and a hybridization and elongation step at 60°C for 30 s, and finally a last “melting curve” step with an increase of 0.5°C every 2 s for up to 95°C. The fluorescence was detected and measured using a CFX Connect real-time PCR system (Bio-Rad). The abundance of transcripts was expressed relative to the control condition according to the method described by Pfaffl (2001) after normalization using already described reference genes *18S RIBOSOMAL RNA* (*Hm18SRNA*) (Chen et al., 2015) *HmACTIN* (Peng et al., 2021), as well as two new reference genes developed in this study: *PROTEIN PHOSPHATASE 2A CATALYTIC SUBUNIT ALPHA* (*HmPP2A*) and *ELONGATION FACTOR 1 ALPHA* (*HmEF1a*). Primers used in this paper were designed on the *H. macrophylla* ‘Aogashima-1’ genome (GCA_013391905.1) available on NCBI (https://www.ncbi.nlm.nih.gov/; Nashima et al., 2021). All primer sequences are available as **Supplementary Data** (see **Supplementary Material**).

### 2.9 Statistical analysis

Graphs and tables represent the means ( ± s.e.) of three independent biological replicates. The number of plants per biological replicate is given in each figure and table. Statistical analyses were carried out using R Studio software version 2022.07.0. All tests (ANOVA parametric test and both Kruskal–Wallis and Wilcoxon–Mann–Whitney non-parametric tests) were performed using an alpha risk error of 0.05.

## 3 Results

### 3.1 MS impacts growth and development of *H. macrophylla* plants

**Figure 1:**
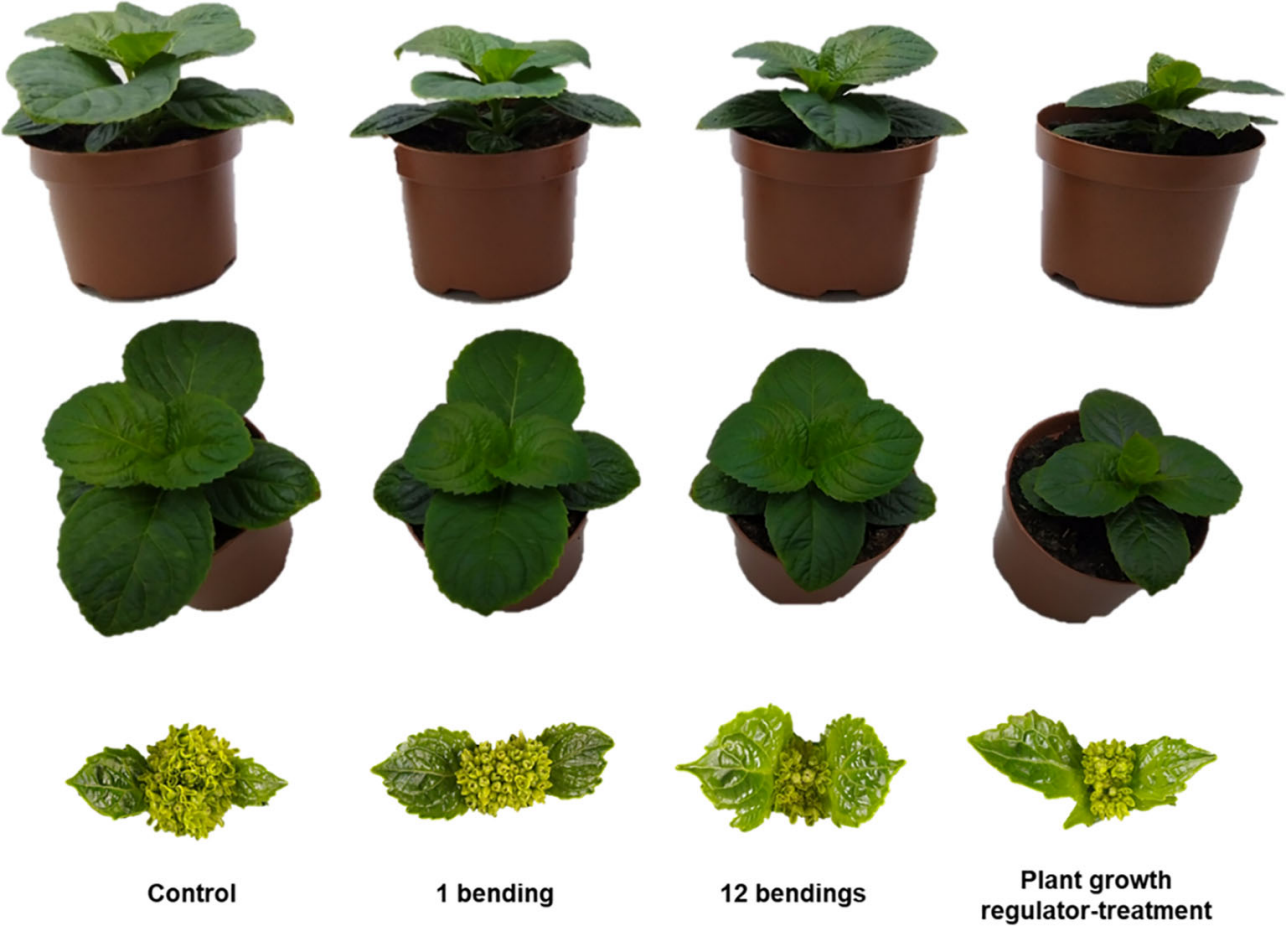
Impact of mechanical stimulation on plant height, leaf growth, and inflorescence development of *Hydrangea macrophylla* after 3 weeks of experiment. Lateral and zenithal views after floral transition of control, mechanically stimulated plants (1 bending/day and 12 bendings/day) and plant growth regulator daminozide-treated plants.

MS treatment caused a significant reduction of stem elongation in young *H. macrophylla* plants. After 3 weeks of MS, stem height was significantly reduced by 38% for the 12B treatment and by 18% for the 1B treatment as compared to the control (**Table 1**). Reduction in stem elongation was already observed during the first week of MS treatment and continued further the following 2 weeks (**Table 1**). Interestingly, the induced percentage of stem elongation reduction was maintained over the time course of 3 weeks for each bending treatment (p > 0.05, **Table 1**): 19%, 7%, and 14% for 1B treatment and 36%, 30%, and 34% for the 12B treatment. This suggests that MS treatment efficiency was constant over the 3 weeks of the experiment. No significant difference in the number of new phytomers produced by MS-treated and control plants was observed (**Table 1**), suggesting that MS does not affect shoot apical meristem (SAM) organogenic activity in *Hydrangea*. The last internode just below the SAM that was still elongating at the start of the experiment (upper preformed internode 3) and both newly formed fourth and fifth internodes were measured at the end of the experiment. The 12B treatment induced a significant reduction in the length of upper preformed internode 3 as well as for the newly formed internodes in comparison to the control (**Table 1**), but such an effect was less visible with the 1B treatment. Together, these results highlight that MS repressed stem elongation in *H. macrophylla* and acts through a reduction of internode elongation, and not through a reduction of SAM organogenesis. MS treatment also promoted the diametrical growth of basal internodes in *H. macrophylla*. The diameter of basal internode 1 was measured at the beginning and end of the 3-week MS treatment. This internode had finished primary elongation and started secondary growth at the beginning of MS treatment, while upper internode 3 pursued elongation during treatment. MS induced an important radial growth at the collar of the stem with an increase of 70% for 1B and 98% for 12B of the internode 1 diameter as compared to the control (**Table 1**). This is confirmed by the measurement of the global area of cross-sections of this internode (**Table 1**). No impact of MS on the diametrical growth of internode 3 was measured (**Table 1**), and no ovalization of the upper and basal internodes occurred under our bilateral MS treatment, while the permanent static flexure did induce an asymmetrical diametrical growth of both internodes. Ovalization was particularly marked in the distal part of the stem with a strong accumulation of cortical tissues on the upper side of the stem (**Supplementary Material**). In addition, under 12B MS treatment, a significant reduction (39%) of leaf expansion occurred (**Table 1**). No such reduction was observed in 1B-stimulated plants. Concerning root development, no difference in the development of the root system after MS was observed (data not shown). Also, when MS plants and control plants were placed in an environment that induces floral transition, no difference in time to flower (bud emergence time point) or in inflorescence sizes was observed (**Figure 1**).

**Table 1:** Comparative impacts of mechanical stimulation (MS) and of plant growth regulator daminozide on stem, internode elongation, and radial growth of *Hydrangea macrophylla* plants.

	Control		MS 1 bending/day		MS 12 bendings/day		Daminozide treatment	
Stem elongation (mm)								
*- After 3 weeks of experiment* *- 1st of experiment* *- 2nd of experiment* *- 3rd of experiment*	27.2 ( ± 1.0) 7.1 ( ± 0.4) 7.8 ( ± 0.6) 12.3 ( ± 0.5)	*a* *a* *a* *a*	21.8 ( ± 1.0) 5.2 ( ± 0.4) 6.4 ( ± 0.4) 10.2 ( ± 0.8)	*b* *b* *a* *b*	16.5 ( ± 0.8) 3.8 ( ± 0.3) 4.6 ( ± 0.3) 8.1 ( ± 0.6)	*c* *c* *b* *b*	10.8 ( ± 0.5) 2.9 ( ± 0.3) 1.6 ( ± 0.2) 6.3 ( ± 0.4)	*d* *d* *c* *c*
Number of internodes	4.9 ( ± 0.2)	*a*	5.0 ( ± 0.2)	*a*	4.9 ( ± 0.2)	*a*	4.5 ( ± 0.2)	*a*
Upper internode 3 elongation (mm)	3.7 ( ± 0.3)	*a*	3.5 ( ± 0.4)	*ab*	2.7 ( ± 0.3)	*b*	1.9 ( ± 0.2)	*c*
Upper internode length (mm)								
*- Internode 4* *- Internode 5*	7.7 ( ± 0.5) 6.1 ( ± 0.5)	*a* *a*	6.6 ( ± 0.4) 4.8 ( ± 0.4)	*a* *a*	5.2 ( ± 0.3) 3.8 ( ± 0.3)	*b* *b*	2.7 ( ± 0.2) 1.7 ( ± 0.1)	*c* *c*
Radial growth of basal internode 1 (mm)	1.2 ( ± 0.08)	*c*	2.1 ( ± 0.11)	*b*	2.4 ( ± 0.11)	*a*	0.9 ( ± 0.06)	*d*
Cross-section area (mm^2^)								
*- Basal internode 1* *- Upper internode 3*	13.6 ( ± 1.3) 17.4 ( ± 0.5)	*c* *a*	20.0 ( ± 1.4) 19.3 ( ± 1.3)	*b* *a*	24.8 ( ± 1.4) 17.9 ( ± 1.2)	*a* *a*	8.6 ( ± 0.5) 11.5 ( ± 0.6)	*d* *b*
Leaf area (cm^2^)	248.6 ( ± 30.3)	*a*	232.6 ( ± 23.2)	*a*	151.9 ( ± 13.1)	*b*	96.0 ( ± 6.7)	*c*

Data are means of three biologically independent replicates, each of 15 plants for stem and internode elongation and radial growth or of five plants for cross-section area and leaf area) ± s.e. Letters indicate significant differences between the different conditions after Kruskal–Wallis non-parametric test (p< 0.05).

### 3.2 MS induces cell wall thickening and lignin accumulation in secondary xylem of basal stimulated internode

**Figure 2:**
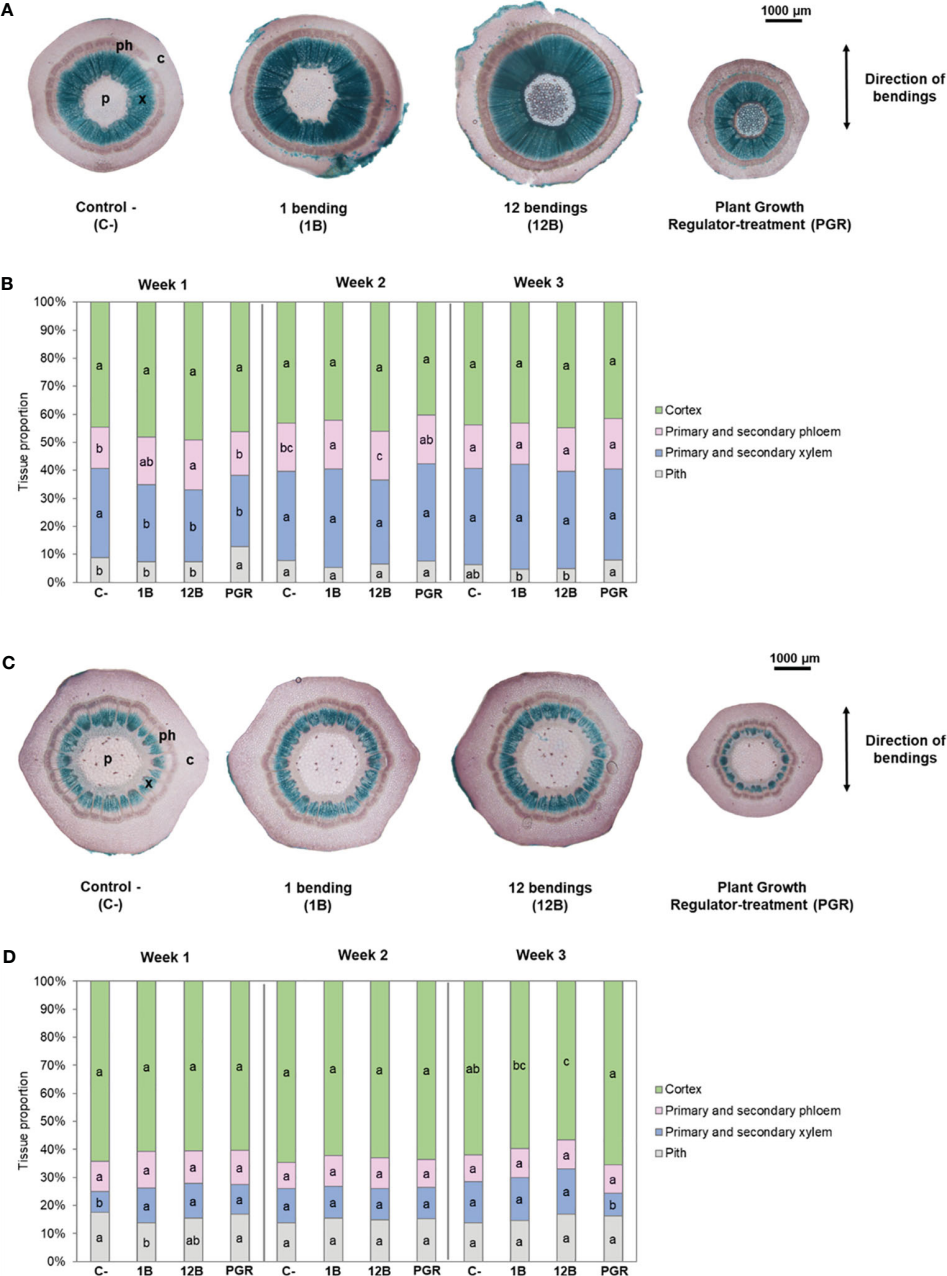
Impact of mechanical stimulation (1B and 12B) and plant growth regulator daminozide on the radial growth of stem internodes in *Hydrangea macrophylla* plants. **(A)** Cross-sections stained with Mirande’s reagent of basal internode 1 at the stem collar and **(C)** in the middle of upper internode 3. **(B)** Relative proportions of the tissues in the cross-sections of the basal 1 and **(D)** of upper internode 3. Data are means of three biologically independent replicates of each of five plants ± s.e. Letters indicate significant differences between the different treatments for the same tissue after Kruskal–Wallis non-parametric test (p< 0.05). p, pith; x, primary and secondary xylem; ph, primary and secondary phloem; c, cortex.

**Figure 3:**
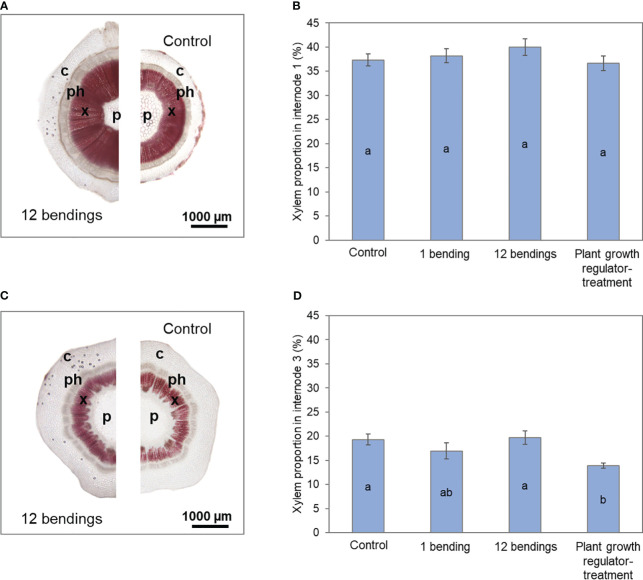
Impact of mechanical stimulation (1 and 12 bendings) and plant growth regulator daminozide on xylem development in internodes of *Hydrangea macrophylla* plants after 3 weeks. **(A)** Cross-sections of basal internode 1 and **(C)** of upper internode 3 stained with Wiesner’s reagent. **(B)** The proportion of xylem tissue in basal internode 1 and **(D)** in upper internode 3. Data are means of three biologically independent replicates of each of two plants ± s.e. Letters indicate significant differences between the different treatments after ANOVA parametric test (p< 0.05). p, pith; x, primary and secondary xylem; ph, primary and secondary phloem; c, cortex.

Histological analysis of cross-sections stained with Mirande’s reagent showed that MS impacted the development of the vascular tissues in the basal internode during the first 2 weeks of treatment (**Figure 2B**). From the first week of treatment, 12B and 1B plants had a slightly lower proportion of xylem and a slightly higher proportion of phloem than control plants. However, after 3 weeks of mechanical treatment, the different proportions of tissues were similar between the stimulated plants (1B and 12B) and the control plants (**Figures 2A, B**, **3A, B**). In upper internode 3, no change in the relative proportions of the different tissues was observed during the 3 weeks of the 1B and 12B treatments (**Figures 2C, D**, **3C, D**).

**Figure 4:**
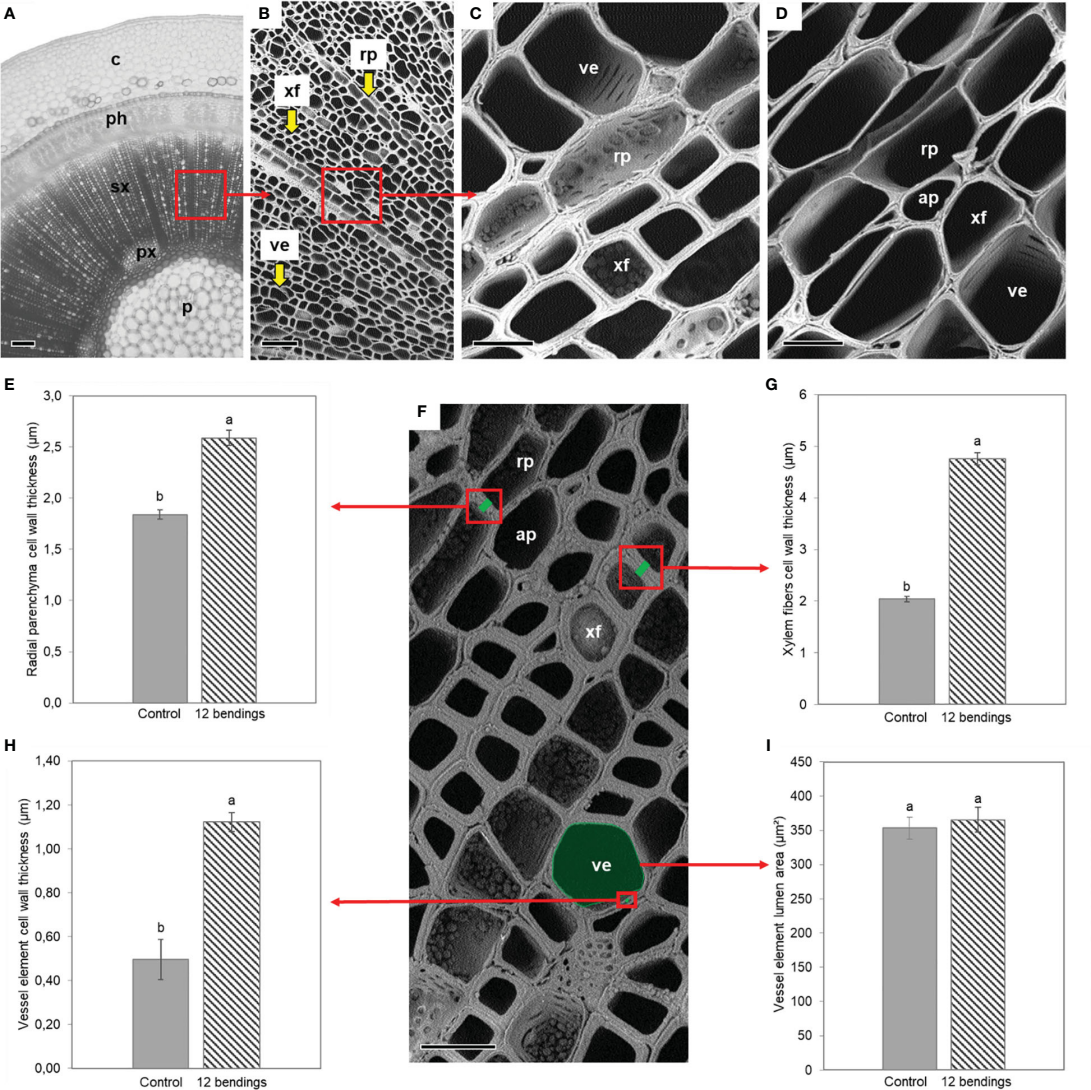
**|** Impact of mechanical stimulation (12 bendings) on xylem cell wall and vessel area in basal internode 1 of *Hydrangea macrophylla* plants after 3 weeks of treatment. **(A)** Light microscopy of cross-sections of basal internode 1. (**B–D**; **F**) Scanning electron microscopy (SEM) of secondary xylem (**C**; **F**) closer SEM in 12B stimulated and **(D)** in control plants. (**E**; **G–I**) Histological analysis. Average cell wall thickness of five pairs of two adjacent cells taken randomly **(E)** in radial parenchyma and **(G)** in wood fibers. **(H)** Average cell wall thickness and **(I)** average lumen area of the five largest vessel elements. Data are means of three independent biological replicates of each of three plants ± s.e. Letters indicate significant differences between the two conditions after Wilcoxon–Mann–Whitney non-parametric test (p< 0.05). p, pith; px, primary xylem; sx, secondary xylem; ph, primary and secondary phloem; c, cortex; xf, xylem fiber; rp, radial parenchyma cell; ap, axial parenchyma cell; ve, vessel element. Scale bars: **(A)** 200 μm, **(B)** 55 μm, **(C, D)** 15 μm, and **(F)** 20 μm.

SEM of the xylem cell wall revealed that the 12B treatment caused a strong cell wall thickening in radial parenchyma cells, xylem fibers, and vessel elements in basal internode 1 (**Figure 4**). Cell walls of xylem fibers and vessel elements were approximately three times thicker than those of the control (**Figures 4G, H**). Results also showed that MS did not induce the development of a cellulosic G-layer in the vessel cell wall, nor a reduction of vessel size in *H. macrophylla* (**Figures 4C, D, I**).

### 3.3 Effects of daminozide in *H. macrophylla*

As expected, treatment using daminozide, one of the usual PGRs used as a dwarfing agent by *H. macrophylla* producers, caused a significant reduction of stem elongation in our experiment (**Table 1**). After daminozide treatment, stems were 56% shorter than those of control plants. Daminozide reached its maximum efficiency only during the second week (72%), and then its efficiency significantly decreased (46%; p< 0.05) during the third week (**Table 1**). Daminozide-treated plants did not increase in stem diameter but rather significantly decreased by 30%. This reduction in diameter was true in both basal internode 1 and upper internode 3 (**Table 1**). However, the low diametral growth of basal internode 1 of daminozide-treated plants was related to the overall reduction in stem growth and development since the proportion of the different tissues composing these internodes was similar to that of control plants (**Figure 2B**). Conversely, the reduction in the cross-section area of upper internode 3 was associated with a lower proportion of xylem tissues (**Figure 2D**). Leaf expansion was also strongly reduced (by 61%) with daminozide treatment (**Table 1**). Finally, daminozide treatment did not impact SAM organogenetic activity or flowering (**Table 1**, **Figure 1**).

### 3.4 Phylogenetic analysis

*H. macrophylla* is a woody species that has been extensively studied for its inflorescence and in particular for its ability to turn blue in the presence of aluminum (Chen et al., 2015; Nashima et al., 2021; Peng et al., 2021; Bak et al., 2021; Guérin et al., 2023; Roman et al., 2023). Recently, the genome of *H. macrophylla* cv. ‘Aogashima-1’ genome was sequenced from young leaves to characterize the double flower phenotype (Nashima et al., 2021). To date, the *Hydrangea* genome is poorly annotated, and so far, very few genes involved in stem development have been studied. Alignment of protein sequences of several model species such as *A. thaliana*, *S. lycopersicum*, *P. trichocarpa*, *C. sinensis*, and *O. sativa* revealed isoforms for 10 candidate genes in *H. macrophylla* (**Supplementary Material**). Eight out of the 10 *H. macrophylla* isoforms had high sequence homology with those of *S. lycopersicum* and *C. sinensis*. *HmXYP1* and *HmPGX3* had the highest homology with respectively *P. trichocarpa* (61%) and *A. thaliana* (89%) sequences (**Supplementary Material**).

### 3.5 MS modulates expressions of cell wall-, cambium-, and lignin biosynthesis-related genes

**Figure 5:**
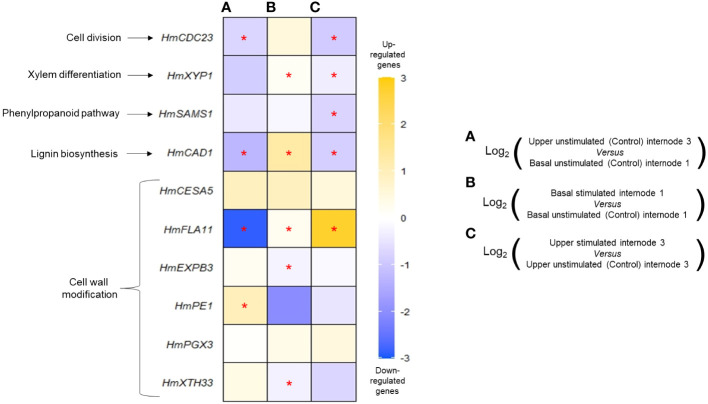
Effects of mechanical stimulation on transcript accumulation of 10 target genes in *Hydrangea macrophylla* internodes. Gene expression was measured 48 h after the last bending of a 2-week treatment with 12 bendings. Transcript accumulation was measured in basal or upper unstimulated or stimulated internodes and compared between them as follows: upper unstimulated internode 3 versus basal unstimulated internode 1 **(A)**, basal stimulated internode 1 versus basal unstimulated internode 1 **(B)**, and upper stimulated internode 3 versus upper unstimulated internode 3 **(C)**. From top to bottom: *HmCDC23* (*CELL DIVISION CYCLE 23*), *HmXYP1* (*XYLOGEN PROTEIN 1*), *HmSAMS1* (*S-ADENOSYL-L-METHIONINE SYNTHETASE 1*), *HmCAD1* (*CINNAMYL ALCOHOL DEHYDROGENASE 1*), *HmCESA5* (*CELLULOSE SYNTHASE 5*), *HmFLA11* (*FASCICLIN-LIKE ARABINOGALACTAN-PROTEIN 11*), *HmEXPB3* (*β-EXPANSINE 3*), *HmPE1* (*PECTINE ESTERASE 1*), *HmPGX3* (*POLYGALACTURONASE INVOLVED IN EXPANSION 3*), and *HmXTH33* (*XYLOGLUCAN: XYLOGLUCOSYL TRANSFERASE 33*). RT-qPCR data used to build the heatmap are available in the **Supplementary Material**. Data are means of n = 3 biological independent replicates ± s.e. Asterisks indicate significant differences between the two conditions after Wilcoxon–Mann–Whitney non-parametric test (p< 0.05).

In order to investigate MS impact at the molecular level, real-time quantitative PCR primers were designed for the *H. macrophylla* isoforms of 10 target genes (**Supplementary Material**), and transcript accumulations measured in basal internode 1 and upper internode 3 were collected 48 h after the last stimulation of a 2-week 12B treatment and in control plants (**Figure 5**). In control plants, analysis revealed a differential expression of some of these genes between upper internode 3 and basal internode 1. In particular, the cell cycle *HmCDC23*, lignin biosynthesis *HmCAD1*, and cell wall regulator *HmFLA11* were downregulated in upper internode 3 in comparison to basal internode 1 (**Figure 5A**). Conversely, the *PECTINE ESTERASE 1* (*HmPE1*) gene was more expressed in upper internode 3 than in basal internode 1 (**Figure 5A**). These differences are likely correlated with the different ongoing processes that take place in these internodes: elongation through primary growth and a start of secondary growth in upper internode 3, and only secondary diametrical growth in basal internode 1. Higher expression of the lignin biosynthesis *HmCAD1* gene measured in basal internode 1 of control plants correlates well with the strong development of the secondary xylem observed in this same internode (**Figure 2A**).

MS caused a deep transcriptional regulation of some of the target genes in the same internodes (**Figures 5B, C**). In basal internode 1 and in comparison with control plants, MS upregulated the xylogenesis *HmXYP1* gene together with the lignin biosynthesis *HmCAD1* and the cell wall modification *HmFLA11* gene. MS also caused a down-expression of cell wall expansion genes *HmEXP3* and *HmXTH33* (**Figure 5B**). In upper internode 3, MS significantly impacted five of the 10 target genes, with three being regulated in basal internode 1 as well, yet with different response patterns. In contrast with basal internode 1, xylem differentiation *HmXYP1* and lignin biosynthesis-related gene *HmCAD1* were significantly repressed by MS in upper internode 3 (**Figure 5C**). Under MS, lower transcript accumulation of the cell division gene *HmCDC23* and *S-ADENOSYL-L-METHIONINE SYNTHETASE 1* (*HmSAMS1*) in upper internode 3 was measured. Interestingly, MS had a stronger promotive effect on the expression of the cell wall *HmFLA11* gene in upper internode 3 than in basal internode 1. Conversely, MS had no impact on *HmCESA5*, *HmPE1*, and *HmPGX3* genes in the *H. macrophylla* stem in the studied internodes.

## 4 Discussion

Little is known about the physiological and molecular processes induced by long-term, recurrent mechanical stimulation, as well as the mechanisms of desensitization. Also, very few plant species have been investigated on these subjects. In this paper, we studied for the first time the responses of the bush *H. macrophylla* to MS and deciphered some histological and molecular changes after 2 and 3 weeks of daily light (1B) or heavy (12B) MS treatments. In addition, we compared responses to MS and chemical plant growth regulator daminozide. When *H. macrophylla* plants were subjected to repetitive symmetrical MS, plants responded to the mechanical loads through a reduction of stem elongation, increased basal diametrical growth, and reduction of leaf expansion (**Table 1**, **Figure 1**). Since the same number of internodes was produced as in control plants, it appears that the reduction in stem height after MS was not due to a repressive effect of the mechanical load on SAM organogenic activity. Rather, stem bending led to a repression of internode elongation, both in those present before treatment and in the most distal internodes that emerged during treatment. According to Coutand et al. (2000), MS impacts not only the stimulated internodes but also the neighboring internodes. These authors demonstrated that the transient bending of the basal part of the tomato stem led to a long-distance effect on the elongating internodes, inducing a growth cessation. In our treatment, where the entire stem was bent by the plastic curtain passing over the plant, each internode was mechanically stimulated, suggesting that the reduced growth of each internode may have been due to direct stimulation. This does not preclude that long-distance signaling after MS between *H. macrophylla* basal and upper internodes may take place and should be investigated further.

Our treatment induced a strong increase in the diametrical growth of *H. macrophylla* stems, up to 98% with the 12B treatment. For this response, not all parts of the stem were affected, but only the most basal internode 1 that had achieved primary growth at the beginning of the treatment. This is consistent with the same differential responses observed along the stem of bell pepper plants when transiently and repetitively bilaterally bent (Graham and Wheeler, 2017). In *A. fraseri* (Telewski, 1989) or hybrid poplar (*Populus tremula* × *Populus alba*, clone INRA 717-1B4) (Roignant et al., 2018), repetitive unilateral flexures caused an ovalization of the stem due to an asymmetrical diametrical growth along the rays subjected to the highest deformation stimuli. In *H. macrophylla*, no asymmetrical growth was observed on bent stems after recurrent bilateral stimulation, even though asymmetrical growth can be produced in this species as illustrated by our experiment imposing permanent unilateral flexure (**Supplementary Material**). Such symmetrical growth of the stem after bilateral MS was also observed in poplar (Kern et al., 2005) and bell pepper stems (Graham and Wheeler, 2017) and suggests that the backward stimulation somehow interacts with the mechanosensitive responses of the forward stimulation.

The higher diametrical growth of the most basal internode 1 of stimulated *H. macrophylla* stem was caused by overall greater development of all tissues with the exception of pith tissues, with its proportion being lower than in control plants (**Figure 3B**). Interestingly, this is different from bean stems, where successive bendings stimulated the development of the pith tissues, allowing the hollow stem of the bean to become more solid and therefore more resistant to MS (Biro et al., 1980). Our results also differ from observation in *A. thaliana*, where mechanical loads on inflorescence stems using a similar mechanical treatment as on *Hydrangea* caused little variation in total stem diameter but were associated with an increase in cortical tissues together with a decrease in lignified and pith tissues (Paul-Victor and Rowe, 2011). These opposite responses may illustrate the different strategies of plants under mechanical constraints according to their growth habits (Niklas and Telewski, 2022): herbaceous plants, such as *A. thaliana* and beans with a short and flexible stem growth strategy, and woody species, such as poplar tree and *Hydrangea* shrub with a short and rigid stem growth strategy for maintaining upright axes in conditions of severe mechanical perturbations (Paul-Victor and Rowe, 2011).

Interestingly, when permanent static flexure of the *H. macrophylla* stem was imposed, the upper internodes, which were still elongating, displayed a stronger asymmetrical development of the cortical tissues than of vascular tissues (**Supplementary Material**). On the contrary, no asymmetrical development of cortical tissues was observed in the basal internode (**Supplementary Material**). This suggests that the herbaceous upper part of the *H. macrophylla* stem reacts to the permanent gravimorphic strain imposed in this experiment through the accumulation of cellulosic tissues rather than through lignified tissues, probably because the expansion of cellulosic cell walls still allows cell and organ elongation. This result also points out that for the same stimulus in the same species, the variability of responses of an organ depends on its developmental stage and needs to be examined carefully.

Our histological analysis demonstrated that the *H. macrophylla* strategy to increase stem stiffness under successive bilateral mechanical constraints is to promote secondary xylogenesis together with a strong thickening of the walls of secondary xylem fibers, parenchyma, and vessels in the basal internode (**Figures 2A**, **3A**, **4C, F**). Only one type of flexure wood developed in the stem cross-sections of *Hydrangea* after bilateral stimulation. On the contrary, in poplar, after successive unilateral mechanical loads, tensile flexure wood with fibers containing a cellulosic G-layer accumulates on the stretched side of the stem, while such fibers were not observed in the compressive flexure wood developing on the other side (Roignant et al., 2018). In *Hydrangea*, only wood fibers without a G-layer formed in the stem after MS. One hypothesis is that under bilateral bending, with both sides of the stem being repetitively stretched and compressed, a unique outcome for developing fibers occurs in *Hydrangea*. This likely corresponds to the best developmental response that allows the *Hydrangea* stem to appropriately withstand recurrent bilateral bendings. This could also imply that the signaling pathway after the stretching stimulus is somehow repressed by the compressive stimulus that follows or, as suggested by Roignant et al. (2018), switched on and off repetitively. Further investigations should bring new information on the dialog between these two developmental pathways and their interactions.

Our molecular data bring first knowledge on the molecular paths controlled by mechanical loads in *Hydrangea* (**Figure 5**). Our results show that *HmFLA11* is upregulated in basal internode 1 of *H. macrophylla* stem upon bending. *HmFLA11* has a high similarity with *AtFLA11* (**Supplementary Material**), a gene suspected to take part in cell surface sensing of mechanical stimulation, the upregulation of which leads to secondary cell wall development (MacMillan et al., 2010; Roignant et al., 2018; Ma et al., 2022a; Ma et al., 2022b; Ma et al., 2023) as we observed in this internode (**Figure 4**). In basal internode 1, the increased development of secondary xylem by MS is also linked to the upregulation of the *XYLOGEN PROTEIN 1* (*HmXYP1*), an arabinogalactan protein that promotes xylem cell differentiation (Motose et al., 2004; Ma et al., 2014). Recently, both *XYP-LIKE PROTEIN*, *XYLP1* and *XYLP2*, were shown to promote lignin biosynthesis during *Capsicum annuum* stem development in response to drought and cold stresses (Zhang et al., 2022). Additionally, the strong accumulation of lignified cells in this secondary wood after MS may be associated with the higher transcription levels of the major lignin synthesis gene *HmCAD1* observed after bendings. Downregulation of *HmEXPB3* and *HmXTH33*, which both promote cell wall extensibility (Yamaguchi et al., 2023), may also contribute, together with the accumulation of lignin, to increasing stem stiffness in the basal part of the bent *Hydrangea* stem.

Our molecular data involved one time point, i.e., 48 h after the last bending of a 2-week treatment. Further transcriptomic studies in *H. macrophylla* will allow us to obtain more knowledge about different actor expressions within a broader time course after flexure. Nevertheless, the late molecular regulations observed 48 h after the last stimulation indicate the long-lasting impact of mechanical stimuli on the molecular machinery. Considering that stems received up to 120 stimulations in 2 weeks and still responded at the transcriptional level to the stimulus, these results point to limited desensitization of *H. macrophylla* stem under our conditions, if not non-existent. This is also illustrated by the constant reduction in stem elongation observed during each of the treatments during 3 weeks. Higher thigmomorphogenic responses under 12B as compared to 1B bending point out as well to a dose response in *H. macrophylla*. This dose response concerns primary and secondary growths of the stem as well as leaf expansion. The relationship between frequencies of mechanical bending and magnitude of growth responses was reported in several species such as *Phaseolus vulgaris* (Jaffe et al., 1980), *Ulmus americana* (Telewski and Pruyn, 1998), and *S. lycopersicum* (Coutand and Moulia, 2000). In *U. americana*, seedlings were significantly shorter with the increasing number of bendings after 3 weeks of experiment. Conversely, no significant differences were observed in diameter growth in the responses to different bending doses, and such responses may indicate an accommodation process of *U. americana* seedlings that is not observed in *H. macrophylla*.

Desensitization to MS is an important response that needs to be evaluated prior to using MS in crop production if an efficient and prolonged dwarfing effect is the aim. We established that 12 bendings per day for five consecutive days per week did not induce such desensitization in *H. macrophylla*, suggesting that at least within this frame, such treatment could be used in crop production. The harmless 12B treatment was two-thirds as effective as the hazardous daminozide treatment in increasing plant compactness (**Table 1**). In contrast to PGR treatment, MS induced a supplemental and important increase of diametrical growth in the basal part of the stem, mainly resulting from greater developments of vascular and cortical tissues (**Figure 2B**). This MS response will benefit plant quality by increasing plant robustness, especially during transplantation and transportation. Additionally, no negative impact of MS was observed on rooting, time to flower, and inflorescence size, which are important criteria for ornamental crop production (**Figure 1**). Thus, our results indicate that MS has strong potential to replace, all or in part, chemical dwarfing molecules in *H. macrophylla* production.

**Figure 6:**
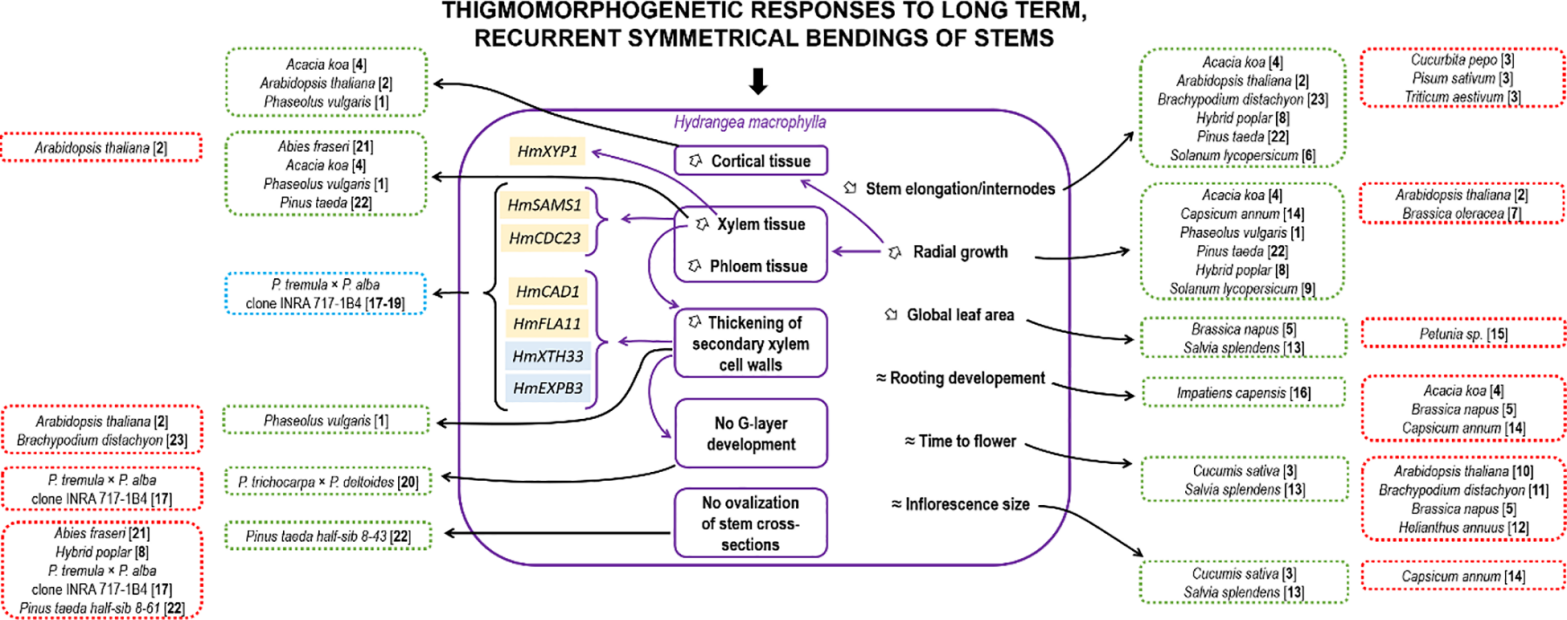
Summary of the main effects induced by long-term, recurrent symmetrical bending in *Hydrangea macrophylla* in comparison with other plant species. Plants have developed patterns of response mechanisms to cope with symmetrical mechanical stress. These mechanisms involve changes to the aerial apparatus, such as a reduction in stem length and/or leaf area, and an increase in stem thickness, notably due to increase in vascular tissues. Changes in gene expression were also observed, but only in species subjected to asymmetrical mechanical stress. Morpho-anatomic and molecular responses of *H. macrophylla* are included in purple boxes. Genes in yellow boxes were found to be significantly upregulated, and those in blue boxes were downregulated in *H. macrophylla* in response to mechanical bending. Green boxes indicate species with common responses with *H. macrophylla*, and red boxes indicate species with opposite responses. Purple arrows link *H. macrophylla* responses that are related to each other. Black arrows link responses in other species. Literature cited: [1] Biro et al., 1980; [2] Paul-Victor and Rowe, 2011; [3] Jaffe, 1973; [4] Ishihara et al., 2017; [5] Cipollini, 1999; [6] Saidi et al., 2009; [7] Biddington and Dearman, 1985; [8] Pruyn et al., 2000; [9] Garner and Björkman, 1996; [10] Lange and Lange, 2015; [11] Gladala-Kostarz et al., 2020; [12] Goodman and Ennos, 1996; [13] Vernieri et al., 2003; [14] Graham and Wheeler, 2017; [15] Jędrzejuk et al., 2020; [16] Anten et al., 2009; [17] Roignant et al., 2018; [18] Pomiès et al., 2017; [19] Martin et al., 2010; [20] Kern et al., 2005; [21] Telewski, 1989; [22] Telewski and Jaffe, 1981; [23] Coomey et al., 2021.

A summary of the thigmomorphogenic effects after long-term, recurrent, symmetrical MS on *H. macrophylla* stems is presented in **Figure 6** with a comparison of other species. For some developmental responses, *H. macrophylla* matches certain species or is opposite to others, independent of their growth habits (ligneous *vs.* herbaceous). Moreover, not one single pattern of responses was observed for all parameters. For example, *H. macrophylla* responses to MS were similar to those of *Brassica napus* for reduction of leaf area but opposite to *B. napus* for root development and time to flower (Cipollini, 1999). Similarly, *H. macrophylla* shares the same responses to MS with *Acacia koa* for stem elongation and diametrical growth but not for root development. This suggests a thin regulation of mechanosensitive responses at the species level and confirms that overall MS impacts on a species, or even a variety (Jędrzejuk et al., 2020), cannot be predicted until assayed. Fewer species were studied for histological responses to recurrent symmetrical bendings. **Figure 6** shows also similar and divergent histological responses in comparison to *H. macrophylla* across species. At the molecular level, only the grass *B. distachyon* has been studied in terms of responses to recurrent, long-term symmetrical bending of the stem, but only root transcriptome modifications were assessed (Coomey et al., 2021). In the roots of this species, several cell wall genes were also regulated after MS loading. Evidence between secondary cell wall thickening after MS and gibberellin inactivation was also provided in *B. distachyon*. Further investigations in *H. macrophylla* should therefore examine the hormonal responses in bent stems after recurrent symmetrical MS to complete this scheme.

## Data availability statement

The datasets presented in this study can be found in online repositories. The names of the repository/repositories and accession number(s) can be found in the article/**Supplementary Material.**

## Author contributions

BL-N: Formal analysis, Investigation, Methodology, Project administration, Validation, Visualization, Writing – original draft, Writing – review & editing. HR: Conceptualization, Funding acquisition, Project administration, Resources, Supervision, Validation, Writing – review & editing. NB: Investigation, Writing – review & editing. LH-T: Conceptualization, Supervision, Validation, Writing – review & editing. VG: Conceptualization, Supervision, Validation, Writing – review & editing. NL: Conceptualization, Funding acquisition, Project administration, Resources, Supervision, Validation, Writing – original draft, Writing – review & editing.
