# Supplementary material for: Recurrent symmetrical bendings cause dwarfing in Hydrangea through spatial molecular regulation of xylem cell walls
Source: Front Plant Sci. 2024 Jan 16;14:1268272. doi: 10.3389/fpls.2023.1268272 (PMC10826399; doi:10.3389/fpls.2023.1268272)
Supplement: Supplementary file 1 [file DataSheet_1.docx]

Supplementary Material

# Supplementary Figures and Tables

## Supplementary Figures

**
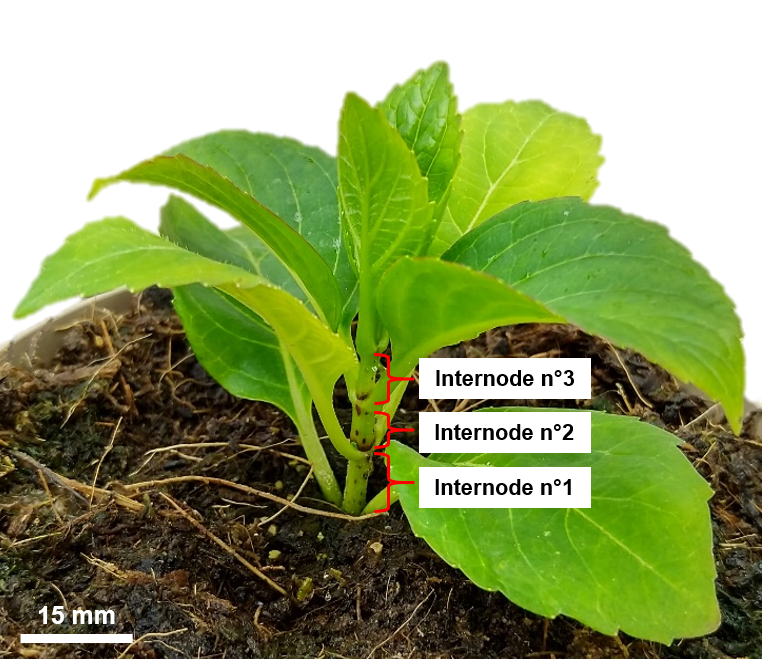
**

**Supplementary Figure 1.** Young *Hydrangea macrophylla* cv. ’Wudu ®’ plant at the beginning of the experiment.

**
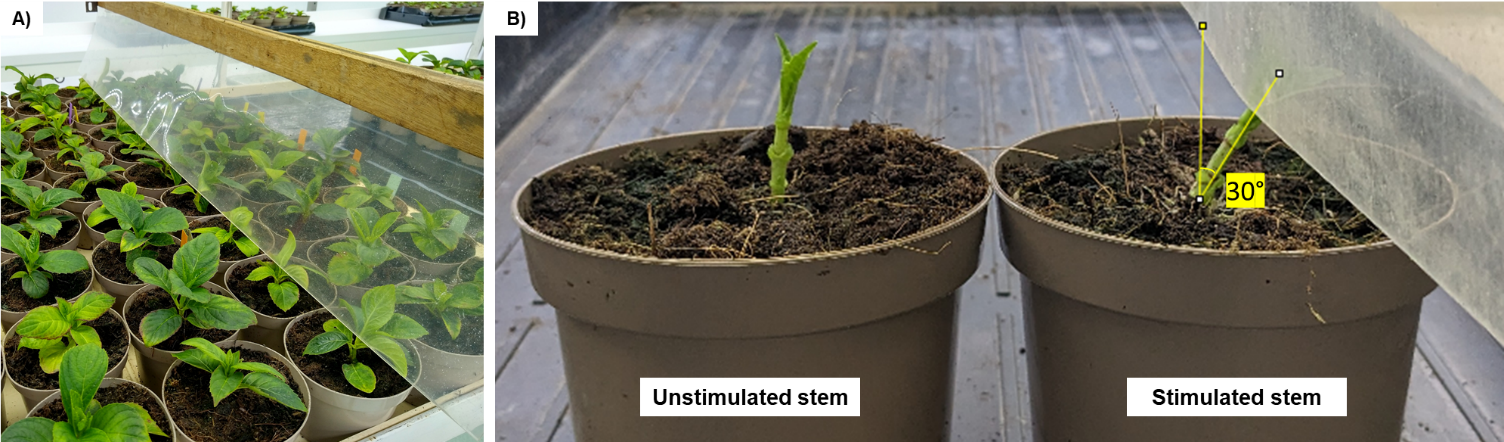
**

**Supplementary Figure 2. (A)** Young *Hydrangea* *macrophylla* cv. ‘Wudu®’ bended by a plastic curtain during mechanical stimulation. **(B)** Bended stem of *H. macrophylla* during mechanical stimulation. Plants were defoliated to facilitate angle measurements here, but not during MS experiments.

**
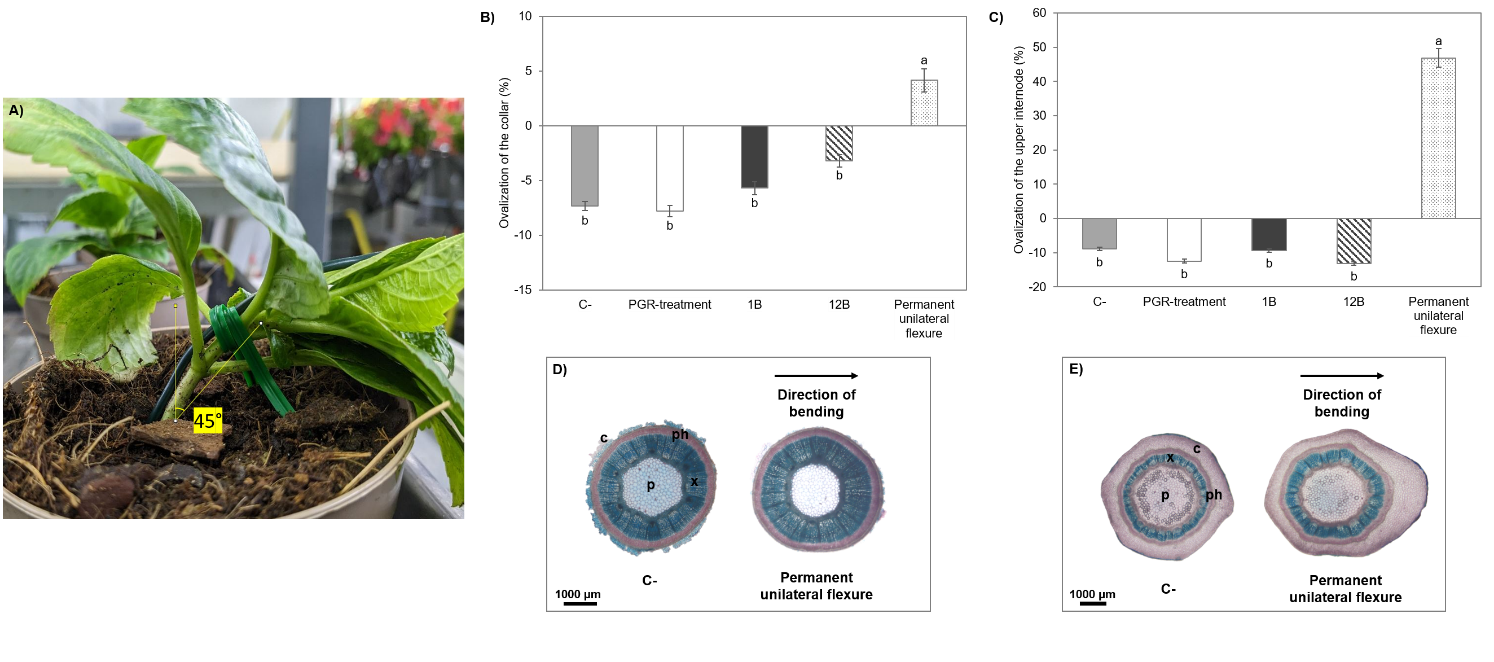
**

**Supplementary Figure 3.** Ovalization of *Hydrangea macrophylla* stem in control (C-), daminozide-treated (PGR-treatment), 1 bending (1B) or 12 bendings (12B) mechanically stimulated and in stems after permanent, static unilateral flexure for 3 weeks. (**A**) Bended stem of *H. macrophylla* during permanent, static unilateral flexure. Percent ovalization in the basal internode (**B**) and in the upper internode (**C**). Cross-sections of the basal internode 1 (**D**) and of the upper internode 3 (**E**) in control (left) and stem under permanent and unilateral flexure (right). A negative ovalization value means that the cross-sections have not undergone eccentric growth, while a positive value indicates an eccentric growth. Data are means of 3 biological independent replicates of 2 plants ± s.e for plants under permanent unilateral flexure and of 5 plants ± s.e for all other conditions. Letters indicate significant differences between treatments after Wilcoxon–Mann–Whitney non-parametric test (P < 0.05). Cortex (c); primary and secondary phloem (ph); primary and secondary xylem (x); pith (p).

**
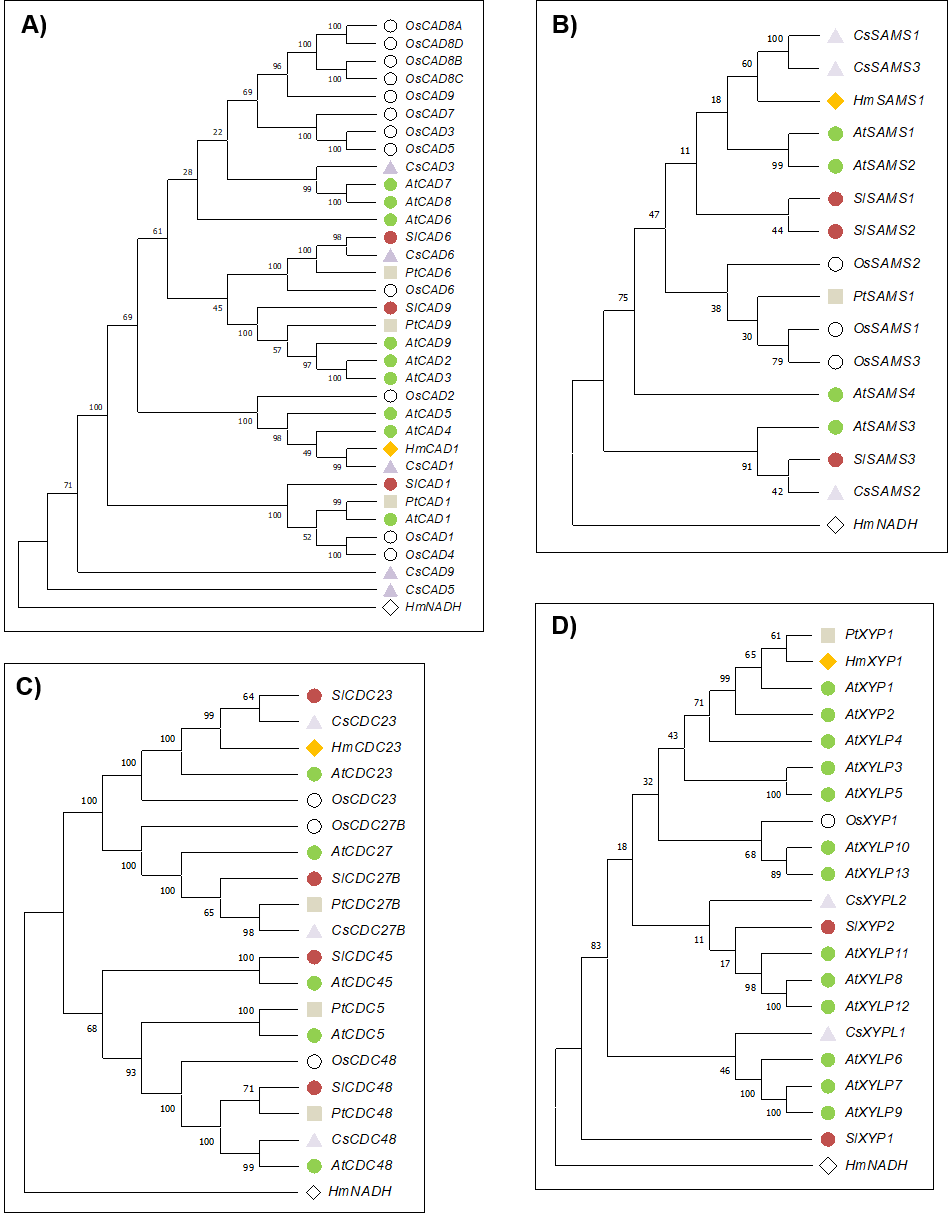
**

**Supplementary Figure 4.** Phylogenetic trees of (**A**) *CAD*, (**B**) *SAMS*, (**C**) *CDC* and (**D**) *XYP* homologs in *H. macrophylla*, *A. thaliana*, *S. lycopersicum*, C. sinensis, *P. trichocarpa* and *O. sativa*. *CAD* (*CINNAMYL ALCOHOL DEHYDROGENASE*); *SAMS* (*S-ADENOSYL-L-METHIONINE SYNTHETASE*); *CDC* (*CELL DIVISION CYCLE*); *XYP* (*XYLOGEN PROTEIN*). The percentage of replicate trees in which the associated taxa clustered together in the bootstrap test (1000 replicates) are shown next to the branches.

**
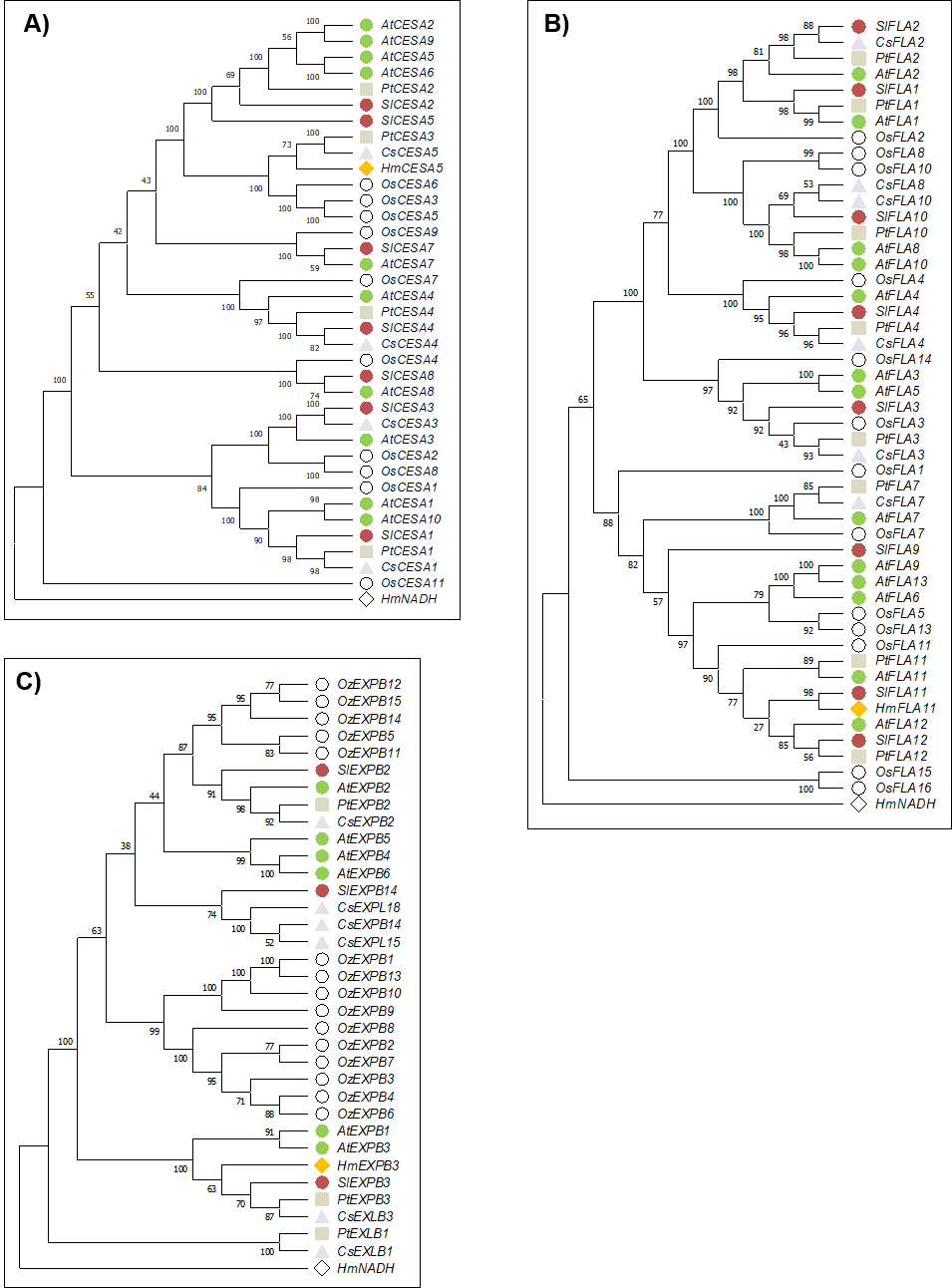
**

**Supplementary Figure 5.** Phylogenetic trees of (**A**) *CESA*, (**B**) *FLA* and (**C**) *EXPB* homologs in *H. macrophylla*, *A. thaliana*, *S. lycopersicum*, *C. sinensis*, *P. trichocarpa* and *O. sativa*. *CESA* (*CELLULOSE SYNTHASE*); *FLA* (*FASCICLIN-LIKE ARABINOGALACTAN PROTEIN*); *EXPB* (*β-EXPANSINE*); *EXLB* (*β-EXPANSINE-LIKE*). The percentage of replicate trees in which the associated taxa clustered together in the bootstrap test (1000 replicates) are shown next to the branches.


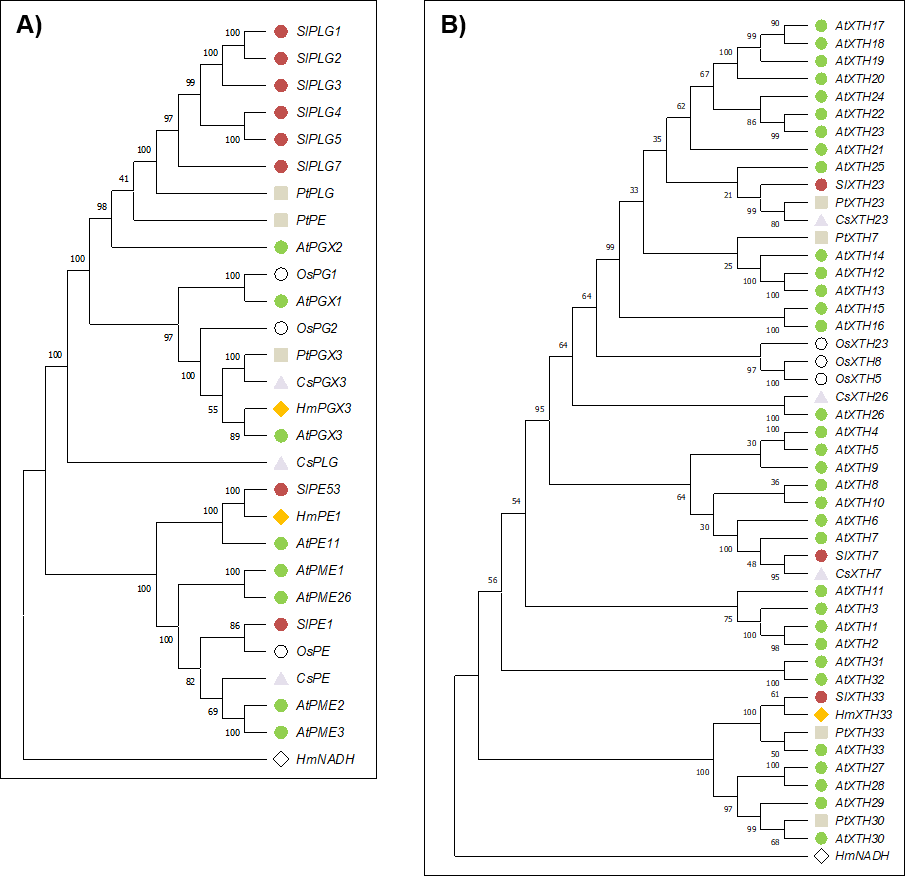


**Supplementary Figure 6.** Phylogenetic trees of (**A**) *PECTINASES* (*PGX*, *PE*) and (**B**) *XTH* homologs in *H. macrophylla*, *A. thaliana*, *S. lycopersicum*, *C. sinensis*, *P. trichocarpa* and *O. sativa*. *PGX* (*POLYGALACTURONASE (PLG) INVOLVED IN EXPANSION*); *PE* (*PECTINE ESTERASE*); *PME* (*PECTINE METHYLESTERASE*); *XTH* (*XYLOGLUCAN:XYLOGLUCOSYL TRANSFERASE*). The percentage of replicate trees in which the associated taxa clustered together in the bootstrap test (1000 replicates) are shown next to the branches.

**
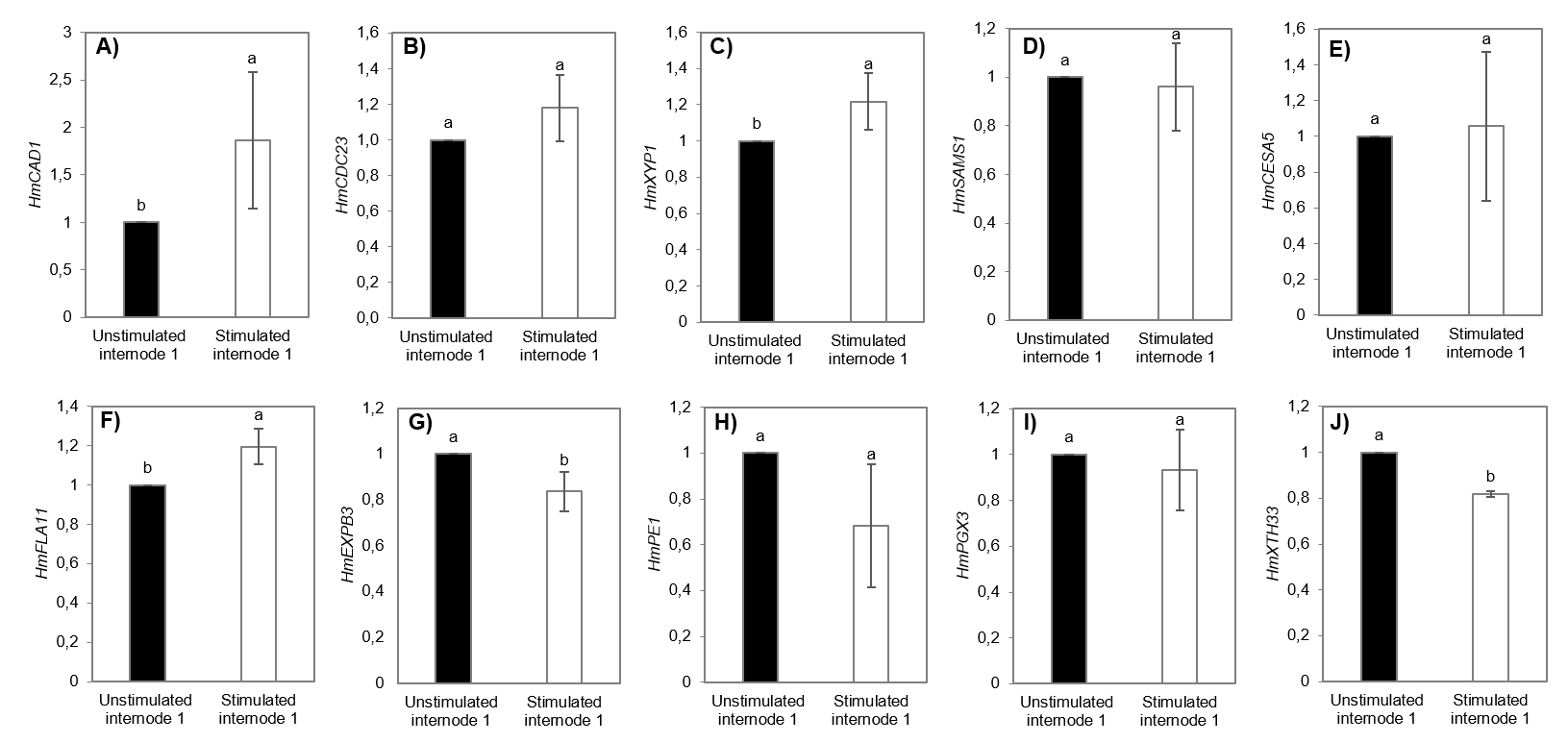
**

**Supplementary Figure 7.** Expression levels of 10 target genes in the basal internode 1 of control and of 12 bendings mechanically stimulated *H. macrophylla* plants after 2 weeks. Tissues were collected 48 hours after the last bending. The expression levels of genes were determined through RT-qPCR. Data are means of n = 3 biological independent replicates ± s.e. The asterisks indicate significant differences after Wilcoxon–Mann–Whitney non-parametric test (P < 0.05).

**
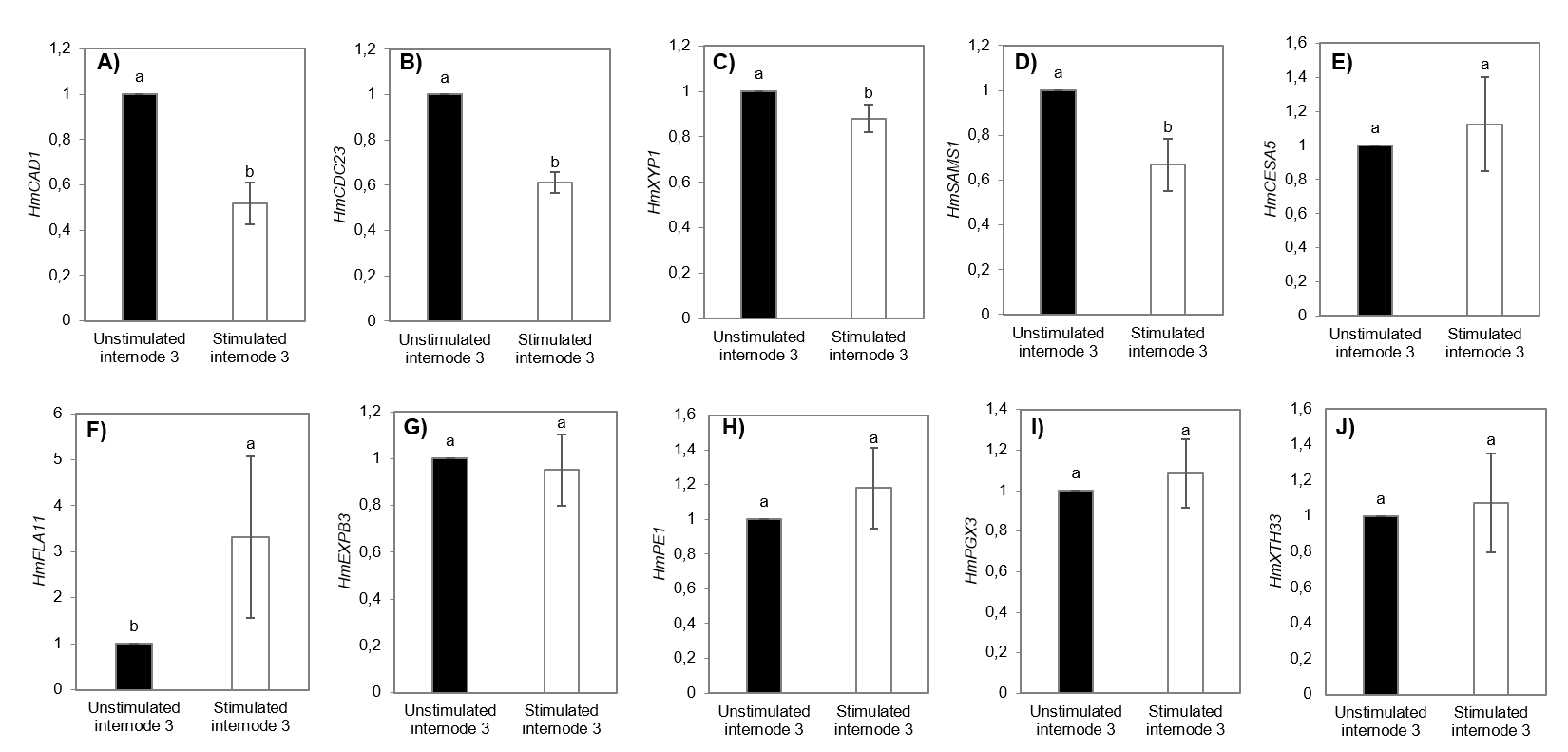
**

**Supplementary Figure 8.** Expression levels of 10 target genes in the upper internode 3 of control and of 12 bendings mechanically stimulated *H. macrophylla* plants after 2 weeks of experiment. Tissues were collected 48 hours after the last bending. Expression levels of genes were determined through RT-qPCR. Data are means of n = 3 biological independent replicates ± s.e. The asterisks indicate significant differences after Wilcoxon–Mann–Whitney non-parametric test (P < 0.05).

**
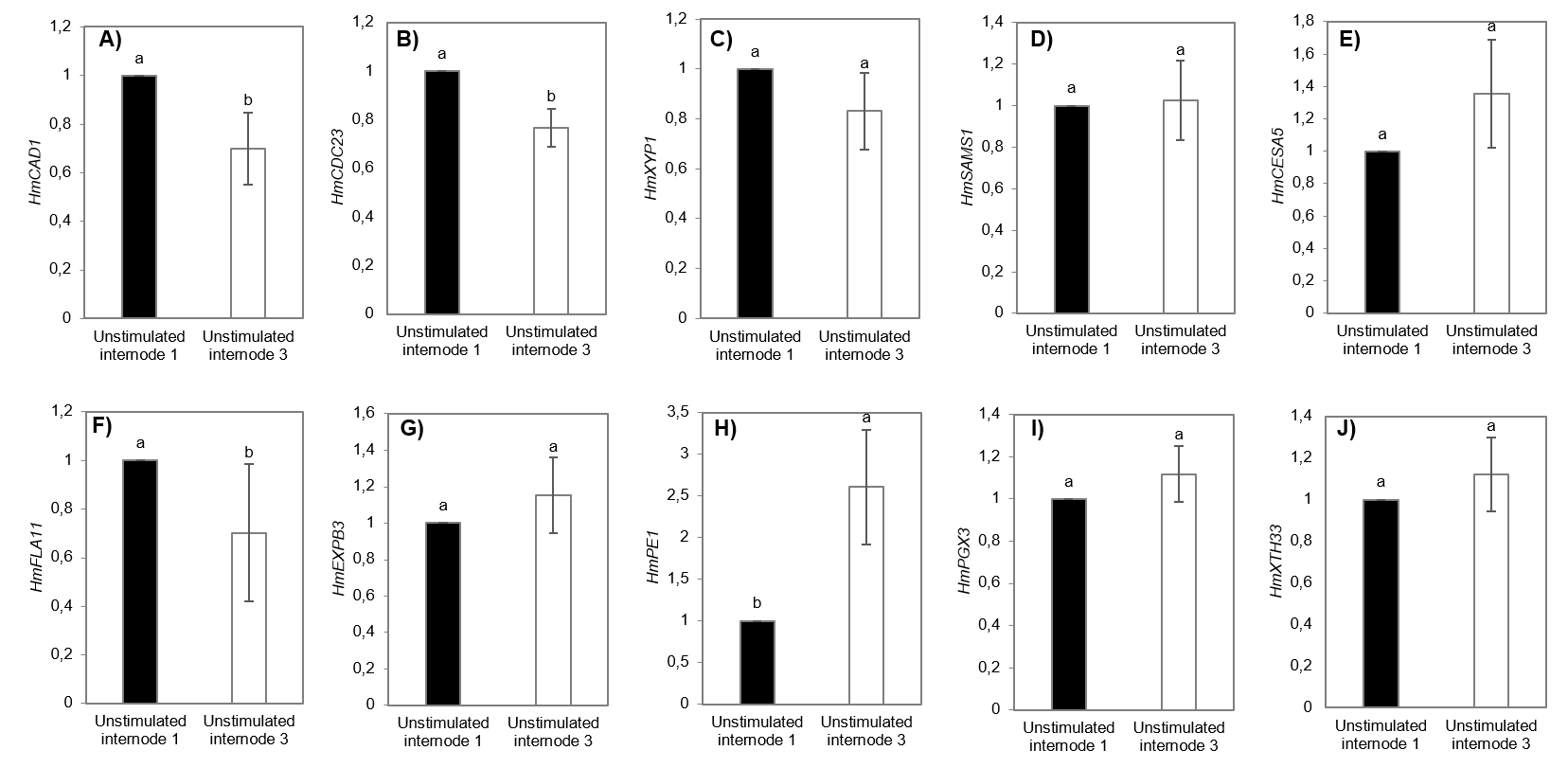
**

**Supplementary Figure 9.** Comparative expression levels of 10 target genes in the basal internode 1 and the upper internode 3 of mechanically stimulated *H. macrophylla* plants 48h after 2 weeks of 12 bendings experiment. Expression levels of genes were determined through RT-qPCR. Data are means of n = 3 biological independent replicates ± s.e. The asterisks indicate significant differences after Wilcoxon–Mann–Whitney non-parametric test (P < 0.05).

## Supplementary Tables

## Supplementary Table 1. Accession number of gene sequences used for phylogenetic trees.

| **Gene ID** | **Name gene** | **Species** | **Gene ID** | **Name gene** | **Species** |
| --- | --- | --- | --- | --- | --- |
| AT1G72680 | *AtCAD1* | *A. thaliana* | AT3G48150 | *AtCDC23* | *A. thaliana* |
| AT2G21730 | *AtCAD2* | *A. thaliana* | AT1G09770 | *AtCDC5* | *A. thaliana* |
| AT2G21890 | *AtCAD3* | *A. thaliana* | AT3G25100 | *AtCDC45* | *A. thaliana* |
| AT3G19450 | *AtCAD4* | *A. thaliana* | AT2G20000 | *AtCDC27B* | *A. thaliana* |
| AT4G34230 | *AtCAD5* | *A. thaliana* | AT3G09840 | *AtCDC48* | *A. thaliana* |
| AT4G37970 | *AtCAD6* | *A. thaliana* | XP_004236154 | *SlCDC23* | *S. lycopersicum* |
| AT4G37980 | *AtCAD7* | *A. thaliana* | XP_004247065 | *SlCDC45* | *S. lycopersicum* |
| AT4G37990 | *AtCAD8* | *A. thaliana* | XP_004234799 | *SlCDC27B* | *S. lycopersicum* |
| AT4G39330 | *AtCAD9* | *A. thaliana* | XP_004236235 | *SlCDC48* | *S. lycopersicum* |
| XP_010314515 | *SlCAD1* | *S. lycopersicum* | XP_028095173 | *CsCDC23* | *C. sinensis* |
| XP_004231578 | *SlCAD6* | *S. lycopersicum* | XP_028122138 | *CsCDC27B* | *C. sinensis* |
| XP_004235066 | *SlCAD9* | *S. lycopersicum* | AFF59215 | *CsCDC48* | *C. sinensis* |
| AEE69007 | *CsCAD1* | *C. sinensis* | BAD25679 | *OsCDC23* | *O. sativa* |
| AEE69008 | *CsCAD3* | *C. sinensis* | XP_015644326 | *OsCDC27B* | *O. sativa* |
| XP_028070413 | *CsCAD5* | *C. sinensis* | XP_015649286 | *OsCDC48* | *O. sativa* |
| XP_028087154 | *CsCAD6* | *C. sinensis* | XP_006381758 | *PtCDC27B* | *P. trichocarpa* |
| XP_028102590 | *CsCAD9* | *C. sinensis* | XP_024438373 | *PtCDC48* | *P. trichocarpa* |
| Q8H859 | *OsCAD1* | *O. sativa* | XP_024439015 | *PtCDC5* | *P. trichocarpa* |
| Q6ZHS4 | *OsCAD2* | *O. sativa* | AT4G32410 | *AtCESA1* | *A. thaliana* |
| Q337Y2 | *OsCAD3* | *O. sativa* | AT4G39350 | *AtCESA2* | *A. thaliana* |
| Q2R114 | *OsCAD4* | *O. sativa* | AT5G05170 | *AtCESA3* | *A. thaliana* |
| Q0J6T3 | *OsCAD5* | *O. sativa* | AT5G44030 | *AtCESA4* | *A. thaliana* |
| Q7XWU3 | *OsCAD6* | *O. sativa* | AT5G09870 | *AtCESA5* | *A. thaliana* |
| Q0JA75 | *OsCAD7* | *O. sativa* | AT5G64740 | *AtCESA6* | *A. thaliana* |
| Q6ERX1 | *OsCAD8A* | *O. sativa* | AT5G17420 | *AtCESA7* | *A. thaliana* |
| Q6ERW9 | *OsCAD8B* | *O. sativa* | AT4G18780 | *AtCESA8* | *A. thaliana* |
| Q6ERW7 | *OsCAD8C* | *O. sativa* | AT2G21770 | *AtCESA9* | *A. thaliana* |
| Q6ERW5 | *OsCAD8D* | *O. sativa* | AT2G25540 | *AtCESA10* | *A. thaliana* |
| Q10PS6 | *OsCAD9* | *O. sativa* | XM_004244983 | *SlCESA1* | *S. lycopersicum* |
| XP_024437027 | *PtCAD1* | *P. trichocarpa* | XM_004237761 | *SlCESA2* | *S. lycopersicum* |
| XP_002301953 | *PtCAD6* | *P. trichocarpa* | XM_004229582 | *SlCESA3* | *S. lycopersicum* |
| XP_002300211 | *PtCAD9* | *P. trichocarpa* | XM_004247452 | *SlCESA4* | *S. lycopersicum* |

**Supplementary Table 1.** Accession number of gene sequences used for phylogenetic trees (continued).

| **Gene ID** | **Name gene** | **Species** | **Gene ID** | | **Name gene** | | **Species** |
| --- | --- | --- | --- | --- | --- | --- | --- |
| XM_026028144 | *SlCESA5* | *S. lycopersicum* | XP_028114703 | *CsXYPL1* | | *C. sinensis* | |
| XM_004242566 | *SlCESA7* | *S. lycopersicum* | XP_028083007 | *CsXYPL2* | | *C. sinensis* | |
| XM_004232976 | *SlCESA8* | *S. lycopersicum* | XP_004238156 | *SlXYP1* | | *S. lycopersicum* | |
| Q6AT26 | *OsCESA1* | *O. sativa* | XP_004235821 | *SlXYP2* | | *S. lycopersicum* | |
| Q84M43 | *OsCESA2* | *O. sativa* | Q6ASY2 | *OsXYP1* | | *O. sativa* | |
| Q69V23 | *OsCESA3* | *O. sativa* | XP_006385219 | *PtXYP1* | | *P. trichocarpa* | |
| Q5JN63 | *OsCESA4* | *O. sativa* | AT1G02500 | *AtSAMS1* | | *A. thaliana* | |
| Q851L8 | *OsCESA5* | *O. sativa* | AT4G01850 | *AtSAMS2* | | *A. thaliana* | |
| Q6YVM4 | *OsCESA6* | *O. sativa* | AT2G36880 | *AtSAMS3* | | *A. thaliana* | |
| Q9AV71 | *OsCESA7* | *O. sativa* | AT3G17390 | *AtSAMS4* | | *A. thaliana* | |
| Q84ZN6 | *OsCESA8* | *O. sativa* | NP_001389410 | *OsSAMS1* | | *O. sativa* | |
| Q69P51 | *OsCESA9* | *O. sativa* | NP_001393225 | *OsSAMS2* | | *O. sativa* | |
| Q69XK5 | *OsCESA11* | *O. sativa* | NP_001396337 | *OsSAMS3* | | *O. sativa* | |
| XP_028108618 | *CsCESA1* | *C. sinensis* | NP_001234425 | *SlSAMS1* | | *S. lycopersicum* | |
| XP_028077069 | *CsCESA3* | *C. sinensis* | NP_001296305 | *SlSAMS2* | | *S. lycopersicum* | |
| XP_028065958 | *CsCESA4* | *C. sinensis* | NP_001304005 | *SlSAMS3* | | *S. lycopersicum* | |
| XP_028066397 | *CsCESA5* | *C. sinensis* | XP_028101546 | *CsSAMS1* | | *C. sinensis* | |
| XP_024459035 | *PtCESA1* | *P. trichocarpa* | XP_028089041 | | *CsSAMS2* | *C. sinensis* | |
| XP_002310628 | *PtCESA2* | *P. trichocarpa* | QGI57475 | | *CsSAMS3* | *C. sinensis* | |
| XP_052308938 | *PtCESA3* | *P. trichocarpa* | AFR41292 | | *PtSAMS1* | *P. trichocarpa* | |
| XP_002301856 | *PtCESA4* | *P. trichocarpa* | AT5G55730 | | *AtFLA1* | *A. thaliana* | |
| AT5G64080 | *AtXYP1* | *A. thaliana* | AT4G12730 | | *AtFLA2* | *A. thaliana* | |
| AT2G13820 | *AtXYP2* | *A. thaliana* | AT2G24450 | | *AtFLA3* | *A. thaliana* | |
| AT4G08670 | *AtXYPL3* | *A. thaliana* | AT3G46550 | | *AtFLA4* | *A. thaliana* | |
| AT5G09370 | *AtXYPL4* | *A. thaliana* | AT4G31370 | | *AtFLA5* | *A. thaliana* | |
| AT1G36150 | *AtXYPL5* | *A. thaliana* | AT2G20520 | *AtFLA6* | | *A. thaliana* | |
| AT1G55260 | *AtXYPL6* | *A. thaliana* | AT2G04780 | *AtFLA7* | | *A. thaliana* | |
| AT2G44300 | *AtXYPL7* | *A. thaliana* | AT2G45470 | *AtFLA8* | | *A. thaliana* | |
| AT4G14815 | *AtXYPL8* | *A. thaliana* | AT1G03870 | *AtFLA9* | | *A. thaliana* | |
| AT2G44290 | *AtXYPL9* | *A. thaliana* | AT3G60900 | *AtFLA10* | | *A. thaliana* | |
| AT3G43720 | *AtXYPL10* | *A. thaliana* | AT5G03170 | *AtFLA11* | | *A. thaliana* | |
| AT2G48130 | *AtXYPL11* | *A. thaliana* | AT5G60490 | *AtFLA12* | | *A. thaliana* | |
| AT3G22600 | *AtXYPL12* | *A. thaliana* | AT5G44130 | *AtFLA13* | | *A. thaliana* | |
| AT2G27130 | *AtXYPL13* | *A. thaliana* | XP_004244059 | *SlFLA1* | | *S. lycopersicum* | |

**Supplementary Table 1.** Accession number of gene sequences used for phylogenetic trees (continued).

| **Gene ID** | **Name gene** | **Species** | **Gene ID** | **Name gene** | | **Species** |
| --- | --- | --- | --- | --- | --- | --- |
| XP_010323900 | *SlFLA2* | *S. lycopersicum* | XP_002318877 | *PtFLA12* | | *P. trichocarpa* |
| XP_010324668 | *SlFLA3* | *S. lycopersicum* | AT1G53840 | *AtPME1* | | *A. thaliana* |
| XP_004236212 | *SlFLA4* | *S. lycopersicum* | AT1G53830 | *AtPME2* | | *A. thaliana* |
| XP_004243817 | *SlFLA9* | *S. lycopersicum* | AT3G14310 | *AtPME3* | | *A. thaliana* |
| XP_004229828 | *SlFLA10* | *S. lycopersicum* | AT3G14300 | *AtPME26* | | *A. thaliana* |
| XP_004241271 | *SlFLA11* | *S. lycopersicum* | AT2G21610 | *AtPE11* | | *A. thaliana* |
| XP_004246667 | *SlFLA12* | *S. lycopersicum* | XP_015637427 | *OsPE* | | *O. sativa* |
| XP_028115805 | *CsFLA2* | *C. sinensis* | NP_001234151 | *SlPE1* | | *S. lycopersicum* |
| XP_028113112 | *CsFLA3* | *C. sinensis* | XP_004251642 | *SlPE53* | | *S. lycopersicum* |
| XP_028096664 | *CsFLA4* | *C. sinensis* | XP_052309727 | *PtPE* | | *P. trichocarpa* |
| XP_028070057 | *CsFLA7* | *C. sinensis* | XP_028069975 | *CsPE* | | *C. sinensis* |
| XP_028083688 | *CsFLA8* | *C. sinensis* | AT3G26610 | *AtPGX1* | | *A. thaliana* |
| XP_028117085 | *CsFLA10* | *C. sinensis* | AT1G78400 | *AtPGX2* | | *A. thaliana* |
| XP_025882038 | *OsFLA1* | *O. sativa* | AT1G48100 | *AtPGX3* | | *A. thaliana* |
| XP_015611848 | *OsFLA2* | *O. sativa* | AAC28903 | *SlPLG1* | | *S. lycopersicum* |
| XP_015624947 | *OsFLA3* | *O. sativa* | AAC28904 | *SlPLG2* | | *S. lycopersicum* |
| XP_015638227 | *OsFLA4* | *O. sativa* | AAC28902 | *SlPLG3* | | *S. lycopersicum* |
| XP_015624844 | *OsFLA5* | *O. sativa* | AAC28905 | | *SlPLG4* | *S. lycopersicum* |
| XP_015648760 | *OsFLA7* | *O. sativa* | AAC28906 | | *SlPLG5* | *S. lycopersicum* |
| XP_015634047 | *OsFLA8* | *O. sativa* | AAC70951 | | *SlPLG7* | *S. lycopersicum* |
| XP_015628294 | *OsFLA10* | *O. sativa* | BAD88298 | | *OsPG1* | *O. sativa* |
| XP_015638718 | *OsFLA11* | *O. sativa* | BAD54709 | | *OsPG2* | *O. sativa* |
| XP_015638062 | *OsFLA13* | *O. sativa* | XP_006372684 | | *PtPLG* | *P. trichocarpa* |
| XP_025881847 | *OsFLA14* | *O. sativa* | XP_024451537 | | *PtPGX3* | *P. trichocarpa* |
| XP_015647857 | *OsFLA15* | *O. sativa* | XP_028088346 | | *CsPGX3* | *C. sinensis* |
| XP_015632406 | *OsFLA16* | *O. sativa* | XP_028119719 | *CsPLG* | | *C. sinensis* |
| XP_002298869 | *PtFLA1* | *P. trichocarpa* | AT4G13080 | *AtXTH1* | | *A. thaliana* |
| XP_002320524 | *PtFLA2* | *P. trichocarpa* | AT4G13090 | *AtXTH2* | | *A. thaliana* |
| XP_006371803 | *PtFLA3* | *P. trichocarpa* | AT3G25050 | *AtXTH3* | | *A. thaliana* |
| XP_024459316 | *PtFLA4* | *P. trichocarpa* | AT2G06850 | *AtXTH4* | | *A. thaliana* |
| XP_024440100 | *PtFLA7* | *P. trichocarpa* | AT5G13870 | *AtXTH5* | | *A. thaliana* |
| XP_002320736 | *PtFLA10* | *P. trichocarpa* | AT5G65730 | *AtXTH6* | | *A. thaliana* |
| XP_024458728 | *PtFLA11* | *P. trichocarpa* | AT4G37800 | *AtXTH7* | | *A. thaliana* |

**Supplementary Table 1.** Accession number of gene sequences used for phylogenetic trees (continued).

| **Gene ID** | **Name gene** | **Species** | **Gene ID** | **Name gene** | **Species** |
| --- | --- | --- | --- | --- | --- |
| AT1G11545 | *AtXTH8* | *A. thaliana* | XP_024459409 | *PtXTH7* | *P. trichocarpa* |
| AT4G03210 | *AtXTH9* | *A. thaliana* | XP_002324481 | *PtXTH23* | *P. trichocarpa* |
| AT2G14620 | *AtXTH10* | *A. thaliana* | XP_006368348 | *PtXTH30* | *P. trichocarpa* |
| AT3G48580 | *AtXTH11* | *A. thaliana* | XP_002320268 | *PtXTH33* | *P. trichocarpa* |
| AT5G57530 | *AtXTH12* | *A. thaliana* | XP_028099004 | *CsXTH7* | *C. sinensis* |
| AT5G57540 | *AtXTH13* | *A. thaliana* | XP_028097969 | *CsXTH23* | *C. sinensis* |
| AT4G25820 | *AtXTH14* | *A. thaliana* | XP_028076879 | *CsXTH26* | *C. sinensis* |
| AT4G14130 | *AtXTH15* | *A. thaliana* | AT2G20750 | *AtEXPB1* | *A. thaliana* |
| AT3G23730 | *AtXTH16* | *A. thaliana* | AT1G65680 | *AtEXPB2* | *A. thaliana* |
| AT1G65310 | *AtXTH17* | *A. thaliana* | AT4G28250 | *AtEXPB3* | *A. thaliana* |
| AT4G30280 | *AtXTH18* | *A. thaliana* | AT2G45110 | *AtEXPB4* | *A. thaliana* |
| AT4G30290 | *AtXTH19* | *A. thaliana* | AT3G60570 | *AtEXPB5* | *A. thaliana* |
| AT5G48070 | *AtXTH20* | *A. thaliana* | AT1G65681 | *AtEXPB6* | *A. thaliana* |
| AT2G18800 | *AtXTH21* | *A. thaliana* | Q40638 | *OzEXPB1* | *O. sativa* |
| AT5G57560 | *AtXTH22* | *A. thaliana* | O24230 | *OzEXPB2* | *O. sativa* |
| AT4G25810 | *AtXTH23* | *A. thaliana* | Q336T5 | *OzEXPB3* | *O. sativa* |
| AT4G30270 | *AtXTH24* | *A. thaliana* | Q94LR4 | *OzEXPB4* | *O. sativa* |
| AT5G57550 | *AtXTH25* | *A. thaliana* | Q7XT39 | *OzEXPB5* | *O. sativa* |
| AT4G28850 | *AtXTH26* | *A. thaliana* | Q7XCA7 | *OzEXPB6* | *O. sativa* |
| AT2G01850 | *AtXTH27* | *A. thaliana* | Q9LD07 | *OzEXPB7* | *O. sativa* |
| AT1G14720 | *AtXTH28* | *A. thaliana* | Q10T32 | *OzEXPB8* | *O. sativa* |
| AT4G18990 | *AtXTH29* | *A. thaliana* | Q7XCG7 | *OzEXPB9* | *O. sativa* |
| AT1G32170 | *AtXTH30* | *A. thaliana* | Q8H7T4 | *OzEXPB10* | *O. sativa* |
| AT3G44990 | *AtXTH31* | *A. thaliana* | Q6H676 | *OzEXPB11* | *O. sativa* |
| AT2G36870 | *AtXTH32* | *A. thaliana* | Q10G40 | *OzEXPB12* | *O. sativa* |
| AT1G10550 | *AtXTH33* | *A. thaliana* | Q946J4 | *OzEXPB13* | *O. sativa* |
| NP_001234475 | *SlXTH7* | *S. lycopersicum* | Q6H677 | *OzEXPB14* | *O. sativa* |
| XP_004235158 | *SlXTH23* | *S. lycopersicum* | Q7XT40 | *OzEXPB15* | *O. sativa* |
| XP_004238674 | *SlXTH33* | *S. lycopersicum* | Solyc10g008440 | *SlEXPB1* | *S. lycopersicum* |
| NP_001390454 | *OsXTH8* | *O. sativa* | NP_001233766 | *SlEXPB2* | *S. lycopersicum* |
| XP_015641106 | *OsXTH23* | *O. sativa* | XP_004243343 | *SlEXPB3* | *S. lycopersicum* |
| BAD06579 | *OsXTH5* | *O. sativa* | XP_004243861 | *SlEXPB14* | *S. lycopersicum* |
| Q76BW5 | *OsXTH8* | *O. sativa* | XP_028119054 | *CsEXLB1* | *C. sinensis* |

**Supplementary Table 1.** Accession number of gene sequences used for phylogenetic trees (continued).

| **Gene ID** | **Name gene** | **Species** | **Gene ID** | **Name gene** | **Species** |
| --- | --- | --- | --- | --- | --- |
| XP_028071704 | *CsEXLB3* | *C. sinensis* | XP_024463835 | *PtEXLB1* | *P. trichocarpa* |
| XP_028119831 | *CsEXPB2* | *C. sinensis* | XP_002320708 | *PtEXPB2* | *P. trichocarpa* |
| XP_028072058 | *CsEXPB14* | *C. sinensis* | XP_002319359 | *PtEXPB3* | *P. trichocarpa* |
| XP_028108410 | *CsEXPB15* | *C. sinensis* | AAF08174 | *HmNADH* | *H. macrophylla* |
| XP_028108409 | *CsEXPB18* | *C. sinensis* |  |  |  |

**Supplementary Table 2.** RT-qPCR primers used in this study.

| **Name genes** | **Forward primers** | **Reverse primers** | **Sequence position on the *H. macrophylla* genome (GCA_013391905.1) (Forward/Reverse)** |
| --- | --- | --- | --- |
| *HmEXPB3* | ACTTCCATTTGCTCCCGGAG | CACTGAGGTCGAAGTGGGTC | 370630 to 370649 /  370707 to 370726 |
| *HmPE1* | TGCGCTCCTATGAACTTGCA | CAGGGGCACTAGGAAAGCAA | 201871 to 201890 /  201936 to 201955 |
| *HmPGX3* | ACGGTCAGCAACACGGTAAT | TGCCAGTTACTGAACCCGAC | 617766 to 617785 /  617831 to 617850 |
| *HmXTH33* | TGAGTGTTCCACCAGAATGC | TGGAAGTGTCAAAACAGGACGA | 887820 to 887839 /  887891 to 887912 |
| *HmCESA5* | ATTGCAGTCCAGTCCACAATC | TCCATTCTCCTTGCATCAATC | 81694 to 81714 /  81778 to 81798 |
| *HmFLA11* | TTGCTGGCGGTTTGAAACTG | CATATTCGCTCCGACCGACA | 40397 to 40416 /  40521 to 40540 |
| *HmCDC23* | CAGCAACTATATCCAGGCTCAGAT | TCAACTCGGTAAGGATCGTTTCTT | 4272303 to 4272326 / 4272390 to 4272413 |
| *HmCAD1* | TCTTGCATCCGAGTCACGTC | CGTTGGACACATGGGGGTTA | 487691 to 487710 /  487818 to 487837 |
| *HmSAMS1* | TTCTCATCCAGGTACTTTTCAGGG | TTAGGGTCCACACGGTTCTTATTT | 410230 to 410253 /  410330 to 410353 |
| *HmXYP1* | CCGGAAGGTACTTGCTGTTC | TTCAAGCTCACACCCAACTG | 136550 to 136569 /  136634 to 136653 |
| *HmPP2A* | GTGCAAGCCTCTGACGGAG | TGTACATTCCATTCTTCAACGAGA | 922724 to 922742 /  922781 to 922804 |
| *HmEF1a* | TCAAGAACCGGGGCATAACC | ACGTGGGTATGTTGCCTCG | 1193272 to 1193291 / 1193376 to 1193394 |
| *Hm18SRNA*  (Chen et al., 2015) | GGAAGTTTGAGGCAATAACAGG | ATTGCAATGATCTATCCCCATC | 7640 to 7661 /  7511 to 7532 |
| *HmACTIN*  (Peng et al., 2021) | GCCTGCCATGTATGTTGCCATC | CGGAATCCAGCACAATACCAGTTG | 4910515 to 4910536 / 4911242 to 4911260 |
